# Mechanoresponsive Protein Crystals for NADH Recycling in Multicycle Enzyme Reactions

**DOI:** 10.1021/jacs.4c04725

**Published:** 2024-07-05

**Authors:** Reza Yekta, Xu Xiong, Jiaxin Li, Bradley S. Heater, Marianne M. Lee, Michael K. Chan

**Affiliations:** School of Life Sciences & Center of Novel Biomaterials, The Chinese University of Hong Kong, Shatin, Hong Kong S.A.R. 999077

## Abstract

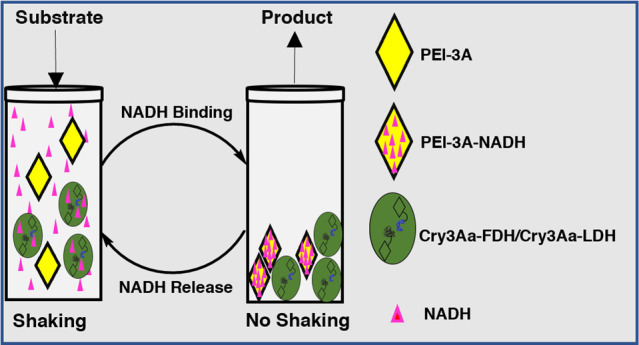

NAD(H)-dependent enzymes play a crucial role in the biosynthesis of pharmaceuticals and fine chemicals, but the limited recyclability of the NAD(H) cofactor hinders its more general application. Here, we report the generation of mechano-responsive PEI-modified Cry3Aa protein crystals and their use for NADH recycling over multiple reaction cycles. For demonstration of its practical utility, a complementary Cry3Aa protein particle containing genetically encoded and co-immobilized formate dehydrogenase for NADH regeneration and leucine dehydrogenase for catalyzing the NADH-dependent l-*tert*-leucine (l-*tert*-Leu) biosynthesis has been produced. When combined with the PEI-modified Cry3Aa crystal, the resultant reaction system could be used for the efficient biosynthesis of l-*tert*-Leu for up to 21 days with a 10.5-fold improvement in the NADH turnover number.

NAD(H)-dependent enzymes exhibit outstanding efficiency and enantioselectivity, making them suitable for multiple applications, particularly the synthesis of pharmaceuticals.^1−5^ The high cost of the cofactor NAD(H), however, poses a challenge for their commercial application.^5−8^ Thus, devising efficient NADH regeneration and recycling strategies is important.

Most approaches for NADH recycling utilize either direct ionic adsorption of the cofactor on the support or its covalent attachment via a linker to the support or the target enzyme. While covalent attachment of the cofactor offers a robust approach that prevents leaching, it suffers from low enzyme activities due to suboptimal interactions between the NADH and enzyme.^9−12^ In contrast, ionic adsorption offers versatility but suffers from cofactor leaching, leading to the eventual loss of NADH over time.^13,14^ Thus, an NADH recycling system that can release and recapture the cofactor without compromising its accessibility to the enzyme would be ideal.

We have developed a mechanoresponsive polyethylenimine-modified Cry3Aa (PEI-3A) crystal that enables the versatile and long-term recycling of NADH in an approach that is simple to operate. The system is distinct in that it utilizes mechanical shaking to promote the reversible release of tightly bound NADH from a NADH storage particle, allowing cofactor-dependent biocatalysts to perform multiple reaction cycles efficiently for up to 21 days. This approach is cost-effective and aligns well with conventional batch reactors, making it suitable for industrial implementation.

We have been exploring the use of Cry3Aa protein crystals produced in *Bacillus thuringiensis* (*Bt*) for multiple applications. The unique biological self-assembly properties of the Cry3Aa protein enable the direct immobilization of functional proteins by genetic fusion or the entrapment of co-expressed protein targets within the Cry3Aa lattice (Figure 1a) in a fashion that stabilizes the immobilized protein against thermal and solvent denaturation.^15,16^ Our interest in the entrapment of cofactors within Cry3Aa crystals was stimulated by the initial observation of NADH binding to Cry3Aa crystals and its positively charged variant, Pos3Aa,^17^ though the amount of NADH binding was deemed inadequate for practical implementation (Figure S1). Given that the more positively charged Pos3Aa crystals bound NADH better than Cry3Aa crystals (11.2 ± 0.7 and 6 ± 2 μmol/g, respectively), we decided to explore the use of protein cationization to improve NADH binding by incorporating the highly positively charged and flexible PEI polymer into the crystal.^18^ In addition to its high cationic charge density, PEI is also attractive because it is inexpensive, is commercially available, and can be easily covalently linked to acidic residues in proteins.^19^

**Figure 1:**
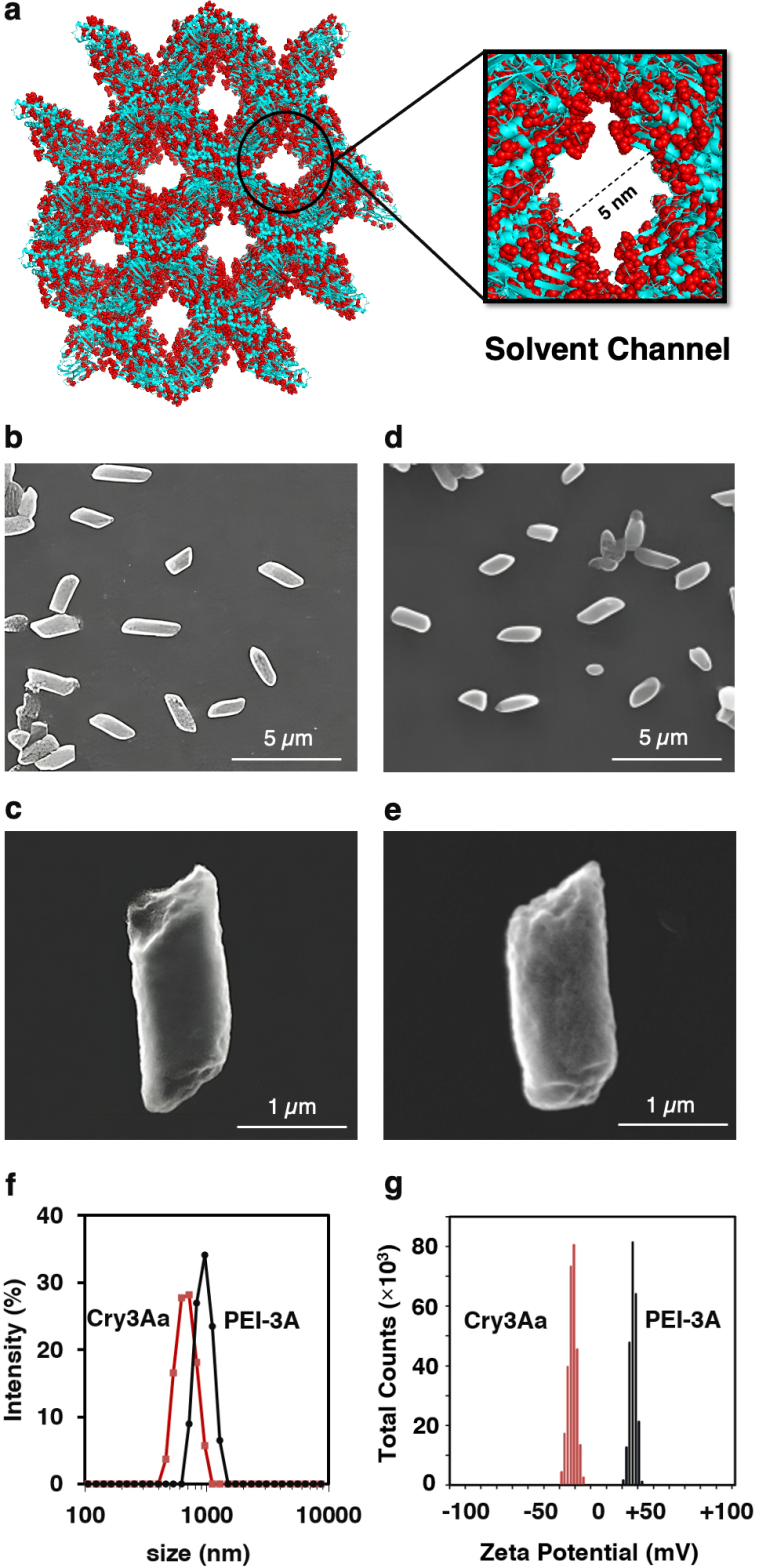
Characterization of PEI-3A crystals. (a) The nanochannels in Cry3Aa crystals (PDB code: 1DLC) are shown in ribbons with Asp and Glu residues highlighted in red and CPK to indicate their distribution. SEM imaging of (b, c) Cry3Aa and (d, e) PEI-3A crystals at low (5000×) and high magnification (150,000×), respectively. (f) Size distribution and (g) zeta potential of Cry3Aa crystals before and after PEI-mediated cationization measured on a Malvern Zetasizer Nano.

PEI-3A crystals were produced by incubating Cry3Aa crystals (Figure S2) with PEI in the presence of EDC/NHS. The successful attachment of PEI could be confirmed based on the ability of copper to form a blue complex with PEI and thus turn the PEI-3A crystals blue (Figure S3). Further confirmation came from confocal microscopy studies showing the binding of Alexa488-labeled PEI (PEI-Alexa 488) to the Cry3Aa crystal (Figure S4). The amount of PEI bound to Cry3Aa crystals was determined to be approximately 12 ± 0.3 mg of PEI per gram of Cry3Aa crystals. Assuming the dimensions of a Cry3Aa crystals are 1.6 μm × 0.80 μm × 0.80 μm,^16^ a single Cry3Aa crystal with 5.5 × 10^6^ Cry3Aa monomers should have ∼80 × 10^3^ PEI molecules distributed within its nanochannels (Supporting Information-Calculations). Analysis of the size distribution of soluble PEI polymers remaining after the PEI-labeling reaction of Cry3Aa crystals suggests that the 5 nm diameter nanochannels of Cry3Aa (Figure 1a, inset) favor PEI polymers smaller than this size (Figure S5).

Based on scanning electron microscopy (SEM) and dynamic light scattering (DLS) of PEI-3A crystals, PEI modification does not alter their rod-like morphology (Figure 1b–e), though a slight increase in the average hydrodynamic diameter was observed for the PEI-3A crystals (914 nm; PDI: 0.102) compared with Cry3Aa’s (825 nm; PDI: 0.08) (Figure 1f). We hypothesize that this slight increase could be due to the covalent attachment of some PEI molecules to the surface of the crystals. As expected, while the original Cry3Aa crystals had a zeta potential of −35 mV, PEI-modification led to a change to +45 mV (Figure 1g). Notably, PEI-cationization of Cry3Aa crystals was highly effective in promoting high levels of NADH binding (540 ± 6 μmol/g) (Figure S6)—an amount that we hypothesized should be sufficient for facilitating NADH-dependent enzyme reactions. Confocal imaging of the PEI-3A crystals treated with NADH showed that the blue fluorescence emitted by NADH permeated the entire crystal (Figure S7). Furthermore, the zeta potential of PEI-3A crystals before and after NADH binding exhibited a significant shift, from +45 to +22 mV, supporting the electrostatic nature of the interaction (Figure S8). This binding was shown to be effective at pH’s near neutral pH and under conditions of low ionic strength (Figures S9–S11).

To elucidate the impact of PEI-cationization on the structure of the Cry3Aa crystals, we took advantage of the fact that *in vitro-*produced Cry3Aa crystals have the same space group and unit cell as the crystals produced in *Bt*.^20,21^ SEM and X-ray crystallographic analyses of the *in vitro*-grown Cry3Aa crystals revealed that the crystal framework was unaffected by PEI-modification (Figures S12 and S13 and Table S1). The crystal structure also gave no evidence of an ordered PEI monomer or specific residue for PEI attachment, suggesting that, while the PEI molecules bind to the Asp and Glu residues within the nanochannels of the crystal, they are anchored randomly. Similar to what was observed with the Cry3Aa crystals produced *in vivo*, confocal microscopy confirmed the labeling of PEI and binding of NADH throughout the *in vitro*-grown PEI-Cry3Aa crystals (Figure 2, Figures S14 and S15, and Movies S1–S3).

**Figure 2:**
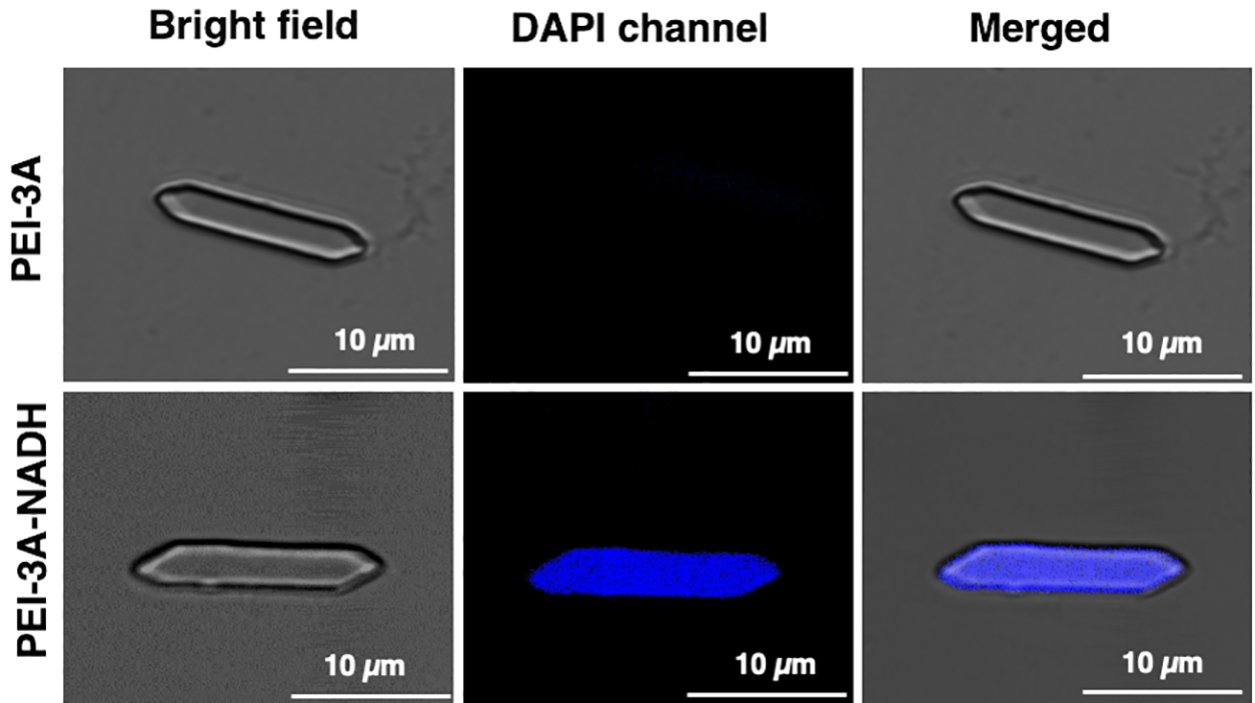
Confocal images of *in vitro*-grown PEI-3A crystals in the presence and absence of NADH. The images show that the bound NADH, which exhibits blue fluorescence in the DAPI channel, is distributed throughout the PEI-3A crystal.

Having characterized the nature of the binding, we then focused on identifying a way to reversibly release the NADH, as such a controlled binding and release would enable the PEI-3A crystals to be used as a storage depot for recycling NADH over multiple enzyme reaction cycles. One viable solution was inspired by previous studies that demonstrated the successful release of molecules from polymers in response to dynamic mechanical environments.^22−25^ These examples prompted us to explore the possibility of regulating the release and binding of cofactor molecules from PEI-3A crystals via the application of mechanical force. Thus, the mechanoresponsiveness of PEI-3A-NADH crystals was investigated by subjecting them to varying levels of orbital shaking (0 to 2000 rpm). These studies revealed that, at 2000 rpm, more than 83 ± 4% of NADH could be released from the PEI-3A crystals, while once the mechanical force ceased (0 rpm), 95 ± 1% of the NADH could be rebound (Figure 3a). This mechanoresponsive release and rebinding process could be repeated across multiple cycles with minimal loss of NADH (Figure 3b), suggesting the potential use of PEI-3A crystals for NADH recycling in batch reactions.

**Figure 3:**
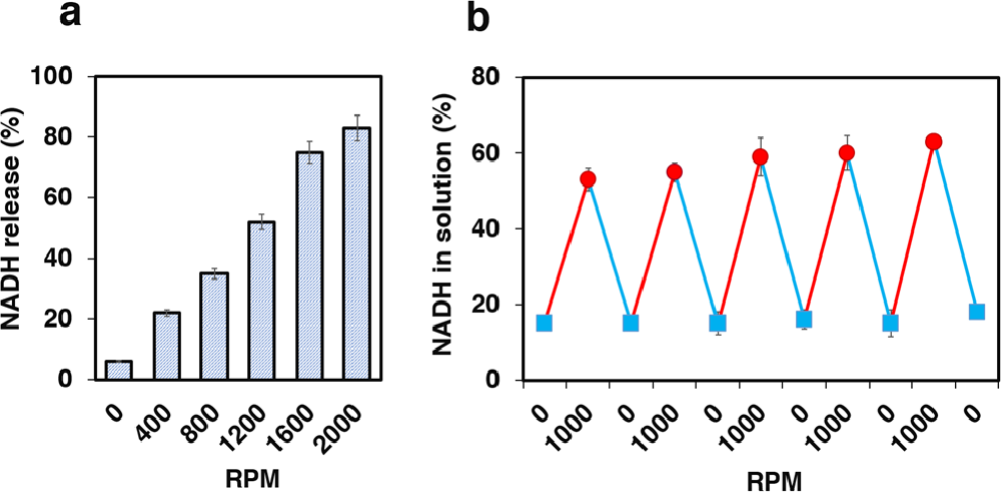
Effect of orbital shaking on the binding and release of NADH within PEI-3A crystals. (a) NADH molecules can be substantially released from PEI-3A crystals by increasing the mechanical agitation. (b) Mechanical-shaking-induced release (red circle) and rebinding (blue square) of NADH within PEI-3A crystals can be repeated for multiple cycles.

The binding affinity of NADH for PEI-3A crystals at different shaking speeds was examined by using binding isotherms and Scatchard analyses. The maximum NADH binding capacity (*B*_max_) of PEI-3A crystals decreased as the orbital shaking speed increased from 0 to 2000 rpm. At 0 rpm, *B*_max_ was 540 ± 6, while, at 2000 rpm, *B*_max_ dropped to 152 ± 5 nmol/mg. Concurrently, the dissociation constant (*K*_d_) increased from 111 ± 1 at 0 rpm to 565 ± 6 μM at 2000 rpm (Figure S16 and Table S2).

To determine the relative force associated with the release of 50% of NADH molecules from the PEI-3A crystals, the following formula was used:
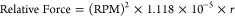
where the relative centrifugal force (RCF) is the radial force generated by the spinning rotor. *r* is the rotation distance (1.5 mm), and RPM is the rotations per minute.^26,27^ According to this formula, it can be estimated that, when the relative force reaches 1.8 mN, 50% of the bound NADH molecules can be detached from PEI-3A crystals into the solution for use in catalysis.

To demonstrate the use of PEI-3A crystals for NADH recycling in a catalytic process, it was necessary to develop a complementary biocatalyst that could effectively utilize NADH to promote a biosynthetic reaction. Building off our previous Cry3Aa-enzyme fusion technologies,^15,16^ we developed a strategy to produce biocatalytic particles containing two protein partners by co-expressing two different Cry3Aa-protein fusions in *Bt.* Our hypothesis was that the Cry3Aa component in the individual Cry3Aa protein fusions might aid in producing co-immobilized fusion particles due to the possibility of their forming similar interactions involved in Cry3Aa crystal nucleation and self-assembly. The feasibility of this approach was tested by co-expressing Cry3Aa-GFP and Cry3Aa-mCherry fusion proteins in *Bt* and characterizing the resulting Cry3Aa-GFP/Cry3Aa-mCherry particles. Fluorescence microscopy was used to show that the resulting particles contained both fluorescent reporter proteins (Figure S17).

With this technology developed, we then applied it to co-immobilize the NAD(H)-dependent enzymes formate dehydrogenase (FDH) and leucine dehydrogenase (LDH) to produce Cry3Aa-FDH/Cry3Aa-LDH particles (Figures S18–S20). Here, FDH was used to promote the regeneration of NADH from NAD^+^ with concomitant oxidation of formate to CO_2_, while LDH was employed to catalyze the conversion of trimethylpyruvate (TMP) and ammonia into l-*tert*-leucine (l-*tert*-Leu) (Figure 4a and Figures S21 and S22).

**Figure 4:**
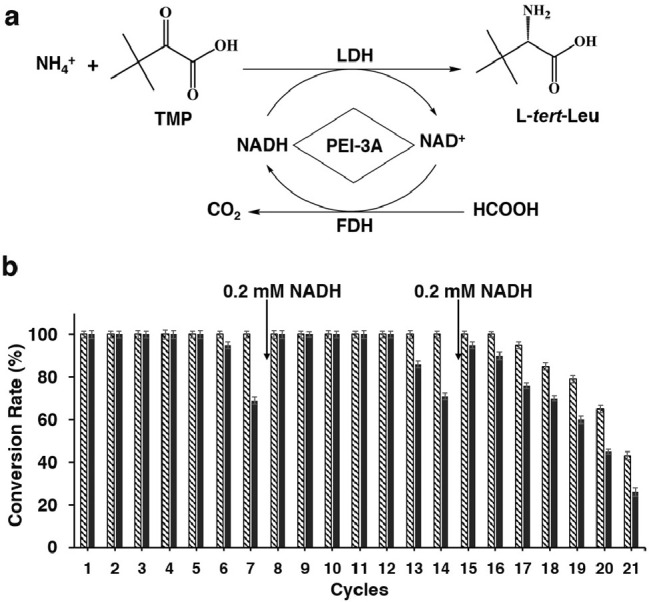
Application of Cry3Aa-FDH/Cry3Aa-LDH particles and PEI-3A crystals for l-*tert*-Leu biosynthesis. (a) Enzymatic conversion of TMP and ammonia to l-*tert*-Leu. (b) Multiple catalytic cycles of Cry3Aa-FDH/Cry3Aa-LDH particles + PEI-3A-NADH crystals (solid bar) and Cry3Aa-FDH/Cry3Aa-LDH particles + 0.5 mM NADH per cycle control (pattern bar) for the production of l-*tert*-Leu.

To probe the impact of immobilization on the enzymes, kinetic studies were performed on the enzymes under turnover conditions (Table S3). These studies revealed that, while the *V*_max_ values of the free and Cry3Aa immobilized enzymes were nearly the same, the *K*_M_ value for the immobilized enzymes was 10-fold higher, suggesting that the loss in activity could be due to conformational changes in the enzymes as a result of immobilization. Although Cry3Aa-mediated immobilization led to a reduction in catalytic functionality of FDH and LDH (Figure S23), the resulting catalyst was stable and recyclable for up to 21 days (Figure 4b), a feature that cannot be achieved by free enzymes.^28,29^

As a case in point, when Lu et al. co-assembled FDH/LDH on a mini scaffold to generate a catalyst for l-*tert*-Leu biosynthesis, both FDH and LDH enzymes exhibited an ∼50% activity reduction after immobilization, and the catalyst could only be used for 3 reaction cycles.^30^ Similarly, in a study by Goa et al., an ∼70% reduction in FDH activity was observed when it was co-immobilized with LDH on polydopamine-coated iron oxide nanoparticles. This led them to generate a mutant FDH that exhibited improved activity, though the catalyst could only perform up to 17 cycles of a 15 min/cycle reaction before enzyme leaching occurred.^31^ In other systems in which the enzymes are not immobilized, such as the one reported by Zhang et al., where FDH and LDH enzymes were fused together using a peptide linker, the enzyme activities were comparable to the free enzymes, which resulted in an improved yield of l-*tert*-Leu that was 1.2-fold higher than the free enzyme mixture. However, no recyclability study was reported, presumably because the system cannot be recycled.^28^

Shaking was found to be important for the catalytic reaction efficiency of the Cry3Aa-FDH/Cry3Aa-LDH particles (Figure S24). Mechanical shaking of the particles increased the yield of TMP produced in the presence of NADH approximately 5-fold without affecting the stability of the particles (Figures S25–S27). These findings highlight the compatibility of Cry3Aa-FDH/Cry3Aa-LDH particles with the release of NAD(H) by the PEI-3A crystals during turnover.

We then combined the reversible NADH-binding properties of PEI-3A crystals with the catalytic properties of Cry3Aa-FDH/Cry3Aa-LDH particles to promote the recyclable production of l-*tert*-Leu over multiple catalytic cycles with minimal NADH supplementation. The mixture of Cry3Aa-FDH/Cry3Aa-LDH particles and NADH-bound PEI-3A crystals exhibited a high conversion rate and the ability to recycle NADH over multiple reaction cycles, although partial (0.2 mM) NADH recharging was required to sustain the conversion efficiency at 100% after 5–7 days (Figure 4b). The activity of the system also gradually declined from cycle 15 onward, reaching 50% activity by cycle 21. The reduction in activity was correlated with a similar loss in activity for the Cry3Aa-FDH/Cry3Aa-LDH particles + 0.5 mM NADH per cycle control reaction run in parallel, suggesting that enzyme degradation was the origin of the loss in activity rather than a reduction in NADH recyclability. The net NADH turnover number (TTN) with PEI-3A-mediated NADH recycling over the 21 days of 495.5 was much higher than the TTN of 46.5 for the reaction with the Cry3Aa-FDH/Cry3Aa-LDH particles + free NADH (Table 1), equating to a 10.5-fold increase in the NADH TTN (Figure S28). This enhancement is significant given that even higher TTN numbers could potentially be achieved if the enzyme stability could be improved.

**Table 1:** Comparison of NADH and LDH Turnover Numbers for the Recyclable Production of l-*tert*-Leu^a^

Catalyst	LDH TTN	NADH TTN
Cry3Aa-FDH/Cry3Aa-LDH + free NADH	9352	46.5
Cry3Aa-FDH/Cry3Aa-LDH + PEI-3A	8544	495.5

a The recyclable production of l-*tert*-Leu by Cry3Aa-FDH/Cry3Aa-LDH particles with and without PEI-3A-mediated NADH recycling was performed for a 21-day period.

This study reveals that PEI-3A crystals serve as an excellent biomaterial for NADH recycling reactions. To evaluate whether this property is associated with the features of the Cry3Aa framework or a property of PEI itself, we evaluated the NADH-binding capacity of a series of related PEI-modified mesoporous silica beads. The key finding was that the NADH binding capacity of PEI-3A crystals is significantly better than any of the PEI-modified silica beads tested (Figures S29 and S31 and Table S4). We speculate that the nanochannels of the Cry3Aa crystals and the nature of the interior of the channel provide an optimal environment for the binding of PEI and, subsequently, NADH.

In summary, PEI-3A crystals have a notable capacity for binding and releasing NADH, making them ideal for aiding in NAD(H)-dependent enzyme reactions. Their mechanical-force-responsive nature allows for the controlled release and rebinding of NAD(H), enhancing their utility as a reservoir. We also report the development of a second technology to efficiently co-immobilize enzymes by taking advantage of the ability of Cry3Aa protein co-expression in *Bt* to generate co-immobilized enzyme particles, such as the Cry3A-FDH/Cry3Aa-LDH particles used in this study. The impact of this breakthrough stems from its potential to provide a simple approach to co-immobilizing multiple enzymes for cascade reactions. Given the simplicity of these approaches and their potential general applicability, we are currently working to demonstrate their applicability to other cofactor-dependent biosynthesis processes.

## Supporting Information Available

Details of supporting results, supporting methods and materials, supporting calculations, and supporting figures and tables (PDF)
Movie S1: *Z*-stacking analysis of PEI molecules within an *in vitro*-grown Cry3Aa crystal (MP4)
Movie S2: *Z*-stacking analysis of NADH immobilization within PEI modified *in vitro*-grown Cry3Aa crystal (MP4)
Movie S3a: The reconstruction of 3D images of a PEI-Alexa488-NADH-Cry3Aa-Alexa568 crystal—the distribution and localization of PEI molecules (green) and NADH molecules (blue) within Cry3Aa crystal (MP4)
Movie S3b: The reconstruction of 3D images of a PEI-Alexa488-NADH-Cry3Aa-Alexa568 crystal—the distribution and localization of Cry3Aa proteins (red) and PEI molecules (green) throughout the crystalline structure (MP4)
