# Supplementary material for: Mechanoresponsive Protein Crystals for NADH Recycling in Multicycle Enzyme Reactions
Source: J Am Chem Soc. 2024 Jul 5;146(28):18817–22. doi: 10.1021/jacs.4c04725 (PMC11258682; doi:10.1021/jacs.4c04725)
Supplement: Supplementary file 1 — ja4c04725_si_001.pdf [file ja4c04725_si_001.pdf]

Supporting Information

Mechanoresponsive Protein Crystals for NADH Recycling in Multicycle Enzyme Reactions

Reza Yekta, Xu Xiong, Jiaxin Li, Bradley S. Heater, Marianne M. Lee, and Michael K. Chan*

School of Life Sciences & Center of Novel Biomaterials, The Chinese University of Hong Kong, Shatin, Hong Kong S.A.R. 999077.

*Corresponding author: Michael K. Chan, E-mail: michaelkchan88@cuhk.edu.hk,

Tel: +852-39431487, Fax: + 852-2603 5646

Table of Contents

Impact of pH and salt concentration on the binding of NADH 5
NADH-binding capacity of PEI-modified mesoporous silica beads 6
Supporting Figures 8
Figure S1. Comparison of the NADH binding capacity of Cry3Aa and Pos3Aa crystals. 8
Figure S2. 10% SDS-PAGE gel of solubilized Cry3Aa crystals.. 9
Figure S3. Colorimetric validation of PEI binding to Cry3Aa crystals. 10
Figure S4. Binding of PEI-Alexa488 to Cry3Aa crystals produced in *Bt*. 11
Figure S5. The size distribution of the PEI polymer before and after anchoring to Cry3Aa. 12
Figure S6. NADH binding capacity of PEI-3A crystals.. 13
Figure S7. Confocal microcopy of NADH-loaded PEI-3A crystals (PEI-3A-NADH).................... 14
Figure S8. Zeta potential analysis of PEI-3A crystals before and after NADH binding. 15
Figure S9. NADH retention by PEI-3A crystals. 16
Figure S10. Impact of pH on NADH binding capacity of PEI-3A crystals.................................... 17
Figure S11. NADH release from PEI-3A crystals as a function of the NaCl concentration. 17
Figure S12. SEM images of *in vitro*-grown crystals. 18
Figure S13. X-ray analysis of PEI-3A crystals. 19
Figure S14. Distribution of PEI molecules within an *in vitro*-grown Cry3Aa crystal. 20
Figure S15. Confocal fluorescence microscopy of NADH-loaded *in vitro*-grown PEI-3A crystals. 21
Figure S16. Determination of B_{max} and K_d. 22
Figure S17. Fluorescence microscopy of particles produced following co-expression of Cry3Aa-GFP and Cry3Aa-mCherry fusion proteins in Bt.. 23
Figure S18. SDS-PAGE analysis of enzyme fusion Cry3Aa particles.. 24
Figure S19. SDS-PAGE analysis of FDH and LDH. 25
Figure S20. SEM images of Cry3Aa-FDH/Cry3Aa-LDH particles. 26
Figure S21. HPLC detection of L-*tert*-Leu modified with OPA. 27
Figure S22. HPLC analysis of the chirality of the *tert*-leu produced by Cry3Aa-LDH enzymes and particles. 28
Figure S23. L-*tert*-Leu production efficiency. 29
Figure S24. The importance of mechanical shaking for L-*tert*-Leu biosynthesis by Cry3Aa-FDH/ Cry3Aa-LDH particles. 30

Figure S25. Stability of Cry3Aa-FDH/Cry3Aa-LDH particles under mechanical agitation........ 31
Figure S26. 10% SDS-PAGE of the supernatant of Cry3Aa-FDH/Cry3Aa-LDH particles after mechanical agitation for a 21-day period. 32
Figure S27. SEM images of Cry3Aa fusion enzyme particles in the absence or presence of mechanical shaking........ 33
Figure S28. The impact of PEI-3A-mediated NADH recycling on the TTN of NADH.. 34
Figure S29. PEI modification of silica beads and subsequent NADH binding. 35
Figure S30. Comparison of NADH binding capacity between PEI-modified silica beads and PEI-3A crystals. 36
Figure S31. Relationship between the amount of PEI anchored to SB1 and its NADH capacity. 36
Supporting Tables 37
Table S1. Data collection and refinement statistics of PEI-3A crystal. 37
Table S2. NADH binding affinity to PEI-3A crystals at different orbital shaking speeds. 38
Table S3. Kinetic parameters of FDH, LDH, and genetically co-immobilized Cry3Aa-FDH/Cry3Aa-LDH particles. 38
Table S4. The density of PEI-3A crystal and silica beads.. 39
Table S5. The list of primers utilized for cloning (5' – 3'). The restriction sites are underlined. 39
Calculations 40
PEI vs Cry3Aa ratio 40
Supporting Methods and Materials 42
Materials 42
Experimental Section 42
Production of PEI-3A crystals 42
Dynamic light scattering (DLS) of Cry3Aa and PEI-3A crystals 43
Binding of NADH to PEI-3A crystals 44
Zeta potential measurement of PEI-3A and Cry3Aa crystals before and after NADH binding .. 44
In vitro growth of Cry3Aa crystals 45
Crystallization, data collection, phase determination and refinement 45
Scanning electron microscope (SEM) of PEI-3A crystals 46
Distribution of PEI and NADH molecules within *in vivo*-grown PEI-3A crystals 46
Size distribution of PEI polymers before and after binding to Cry3Aa crystals 47
Retention of NADH on PEI-3A crystals 47
Effect of pH on NADH binding to PEI-3A Crystals 48
Impact of salt concentration on NADH binding to PEI-3A crystals 48

NADH release from PEI-3A crystals by mechanical shaking 48
Rebinding of NADH molecules into PEI-3A crystals 49
NADH binding to PEI-3A crystals as a function of mechanical agitation speed 49
Cry3Aa mediated co-immobilization of GFP and mCherry proteins 50
Construction of Cry3Aa-FDH and Cry3Aa-LDH expression vectors 50
Cry3Aa NADH-dependent enzyme fusion crystals 51
Expression and purification of soluble FDH and LDH proteins 52
Identification of L-*tert*-Leu by HPLC 53
Determining the chirality of the *tert*-leucine product 53
Determination of enzyme kinetic parameters 54
Effect of mechanical shaking on L-*tert*-Leu production efficiency 54
Stability of Cry3Aa-FDH/Cry3Aa-LDH particles under mechanical agitation 54
Combining PEI-3A-NADH and Cry3Aa-FDH/Cry3Aa-LDH particles for recyclable L-*tert*-leu biosynthesis 55
Determination of the L-*tert*-Leu produced by different LDH and FDH constructs 56
Scanning electron microscopy (SEM) of Cry3Aa-FDH/Cry3Aa-LDH particles 56
Production of PEI-modified mesoporous silica beads 57
References 59

Impact of pH and salt concentration on the binding of NADH

To evaluate the inherent ability of PEI-3A crystals to retain the bound NADH in the absence of the exogenous cofactor, PEI-3A crystals were washed with Na-PB (10 mM, pH 7.0) for multiple cycles. As shown in Figure S9**,** less than 50% of the NADH bound to the PEI-3A crystals was released after 9 cycles of washing with Na-PB, at which point the crystals could be washed without significant loss of NADH for up to cycle 20. These results suggest that PEI-3A crystals have a high capacity for retention of NADH molecules, presumably due to electrostatic interactions between the PEI and NADH molecules in the nanochannels inherent to the Cry3Aa crystal framework. In confirmation of this, washing the NADH-bound PEI-3A crystals with 1M Na-PB (pH 7.0), whose phosphate ions can compete with the NADH for binding to PEI, resulted in the release of nearly all NADH.

Given that PEI-3A crystals bind to NADH via electrostatic interactions, the strength of this interaction was expected to be impacted by pH. To determine the effect of this factor on NADH binding capacity of PEI-3A crystals, Na-PB buffer was chosen for this study as it is a common buffer used in enzymatic reactions and has a wide buffering range, from 5.8 to 8.0. The binding affinities of NADH to PEI-3A crystals in 25 mM NaPB at pH 6.0, 7.0, and 8.0 were evaluated and determined to be 453.5 ± 2.8 µmol/g, 412 ± 4 µmol/g, and 350.2 ± 3.6 µmol/g, respectively (Figure S10). The applicability of PEI-3A crystals for NADH binding in a basic environment was also tested in ammonium formate (25 mM, pH 9-10). While the NADH binding capacity of PEI-3A crystals was calculated to be 134± 3.1 and 42± 5 µmol/g at pH 9.0 and 10.0, respectively (Figure S10).While there is a general

decrease in the binding affinity with increasing pH, presumably due to the deprotonation of the PEI amines, PEI-3A still retains some NADH binding capacity at these higher pH's.

To evaluate the impact of ionic strength more quantitatively, the binding affinity of NADH to PEI-3A crystals was determined by measuring the release of NADH molecules from PEI-3A-NADH crystals at a fixed concentration of NaPB (25 mM, pH 7.0) but a range of concentrations of NaCl (0-800 mM) (Figure S11). As the NaCl concentration increased, the percentage of NADH released from NADH-bound PEI-3A crystals increased from 5 ± 2.4% to 94 ± 5.3%. These results confirm that NADH binding to PEI-3A crystals is significantly affected by the salt concentration. Notably, however, while high concentrations of salt can be used to release NADH from the crystal, at the lower salt concentrations commonly used with proteins (e.g. 50 mM NaCl), the retention of NADH remains quite high.

NADH-binding capacity of PEI-modified mesoporous silica beads

To explore the effect of solid support on the NADH binding capacity, we prepared PEI-modified silica beads (PEI-SBs) with different bead and pore sizes, including PEI-SB1 (1 µm beads with 4 nm pores), PEI-SB2 (1 µm beads with 10 nm pores), and PEI-SB3 (150 nm size with 4 nm pores), and characterized their NADH binding capacity. As shown in Figure S29, PEI-Alexa 488 molecules were found to bind efficiently to the micrometer-sized silica beads based on the high levels of fluorescence observed. These PEI-Alexa488-silica beads were then incubated with NADH to allow for entrapment of the NADH within the beads. The binding of NADH within the beads was visually confirmed based on the fluorescence observed in the DAPI channel (Figure. S29).

To gain insight into the impact of PEI-SB particle size and pore diameter on NADH binding, we prepared each of the three types of PEI-SB with the same amount of bound PEI. The results revealed that the PEI-SB1 beads (60 ± 1.3 µmol/g) with the smaller pore size, exhibited a two-fold higher affinity than that of the PEI-SB2 beads (32 ± 2.4 µmol/g) (Figure S30). This tighter binding may be due to the smaller pore size inducing a more confined space for NADH binding, leading to a stronger interaction with the PEI. On the other hand, comparison of PEI-SB1 with PEI-SB3, having the same 4 nm pore size but a smaller bead size, revealed that NADH binds with 1.9-fold higher affinity to PEI-SB3 (114 ± 0.9 µmol/g) (Figure S30). Our hypothesis is that PEI is better able to penetrate into the smaller bead, and thus for the same weight of bead, there is more PEI bound and thus more sites to bind the NADH, leading to a higher immobilization capacity.

To study the impact of PEI concentration on the NADH binding capability of silica beads (SB), we chose PEI-SB1, which has similar pore size characteristics to PEI-3A crystals. As shown in Figure S31, increasing the quantity of PEI (0-65 mg PEI/g of SB) linked to the beads resulted in a significant increase in the amount of NADH bound from 0 to 105 ± 1.2 µmol/g. However, after the amount of bound PEI reached a threshold of 60 ± 1 mg PEI/g of silica beads, the NADH binding capacity decreased. These findings may suggest that increasing the concentration of PEI used to label the SB beads leads to higher levels of anchored PEI and, in turn, a larger quantity of NADH being bound. However, beyond a certain point (i.e. ~ 60 mg per g of SB1 beads), further increases in the amount of PEI loaded lead to a decrease in NADH binding capacity. This could be due to PEI overcrowding, which leads to decreased space for the NADH molecules to fit.

Supporting Figures

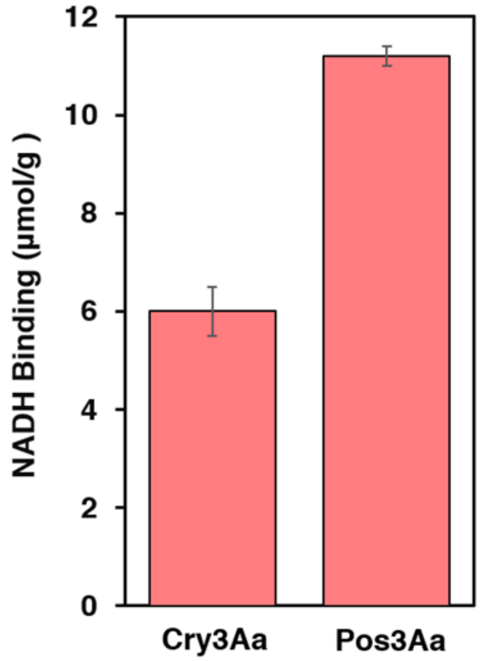

Figure S1. Comparison of the NADH binding capacity of Cry3Aa and Pos3Aa crystals.

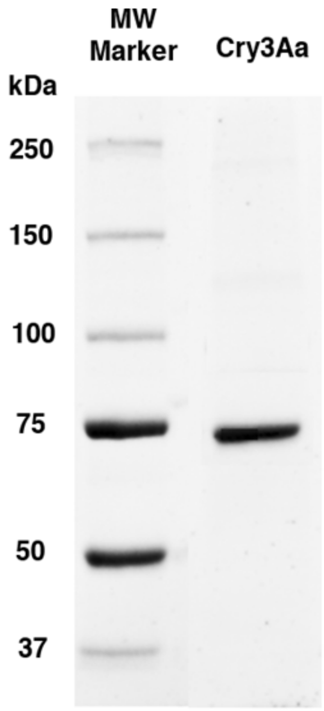

Figure S2. 10% SDS-PAGE gel of solubilized Cry3Aa crystals. The gel clearly shows a prominent band at 73 kDa, signifying the high purity of the produced Cry3Aa.

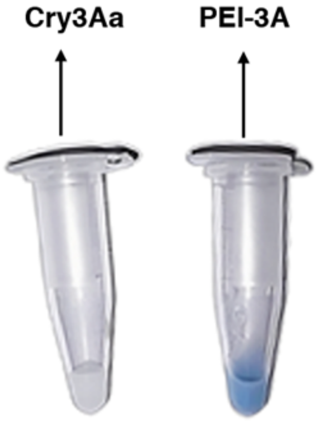

Figure S3. Colorimetric validation of PEI binding to Cry3Aa crystals. PEI binding to Cry3Aa crystals could be confirmed by the ability of PEI to bind to Cu^{2+} ions to produce a dark blue PEI-Cu^{2+} complex[1]. When PEI-3A crystals were treated with cupric sulfate, the crystals immediately turned from white to dark blue, while untreated Cry3Aa crystals mixed with cupric sulfate solution remained white.

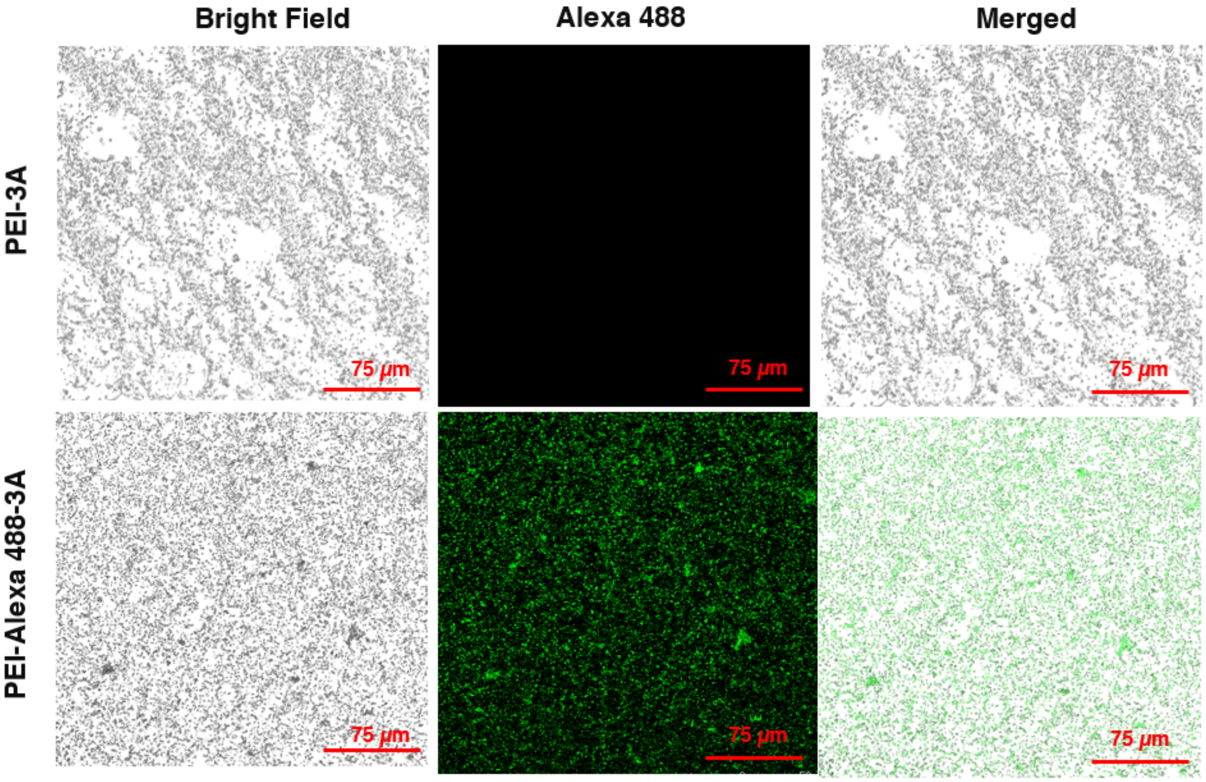

Figure S4. Binding of PEI-Alexa488 to Cry3Aa crystals produced in *Bt*. The binding of PEI polymer within *in vivo*-produced Cry3Aa crystals was assessed by first labeling the PEI polymer with Alexa Fluor™ 488 NHS Ester in 100 mM HEPES buffer (pH 7.5). The PEI solution was washed with 100 mM HEPES buffer using a centrifugal filter (3 kDa, Amicon® Ultra) multiple times to remove unbound Alexa 488 molecules. Then, PEI-Alexa 488 polymers were bound to Cry3Aa crystals in the presence of EDC/NHS cross-linker agents. After multiple washing cycles of the crystals with NaCl (100 mM) and ddH_2O, the crystals were examined by confocal fluorescence microscopy. The observed data indicates that PEI-Alexa488 is effectively bound with Cry3Aa crystals.

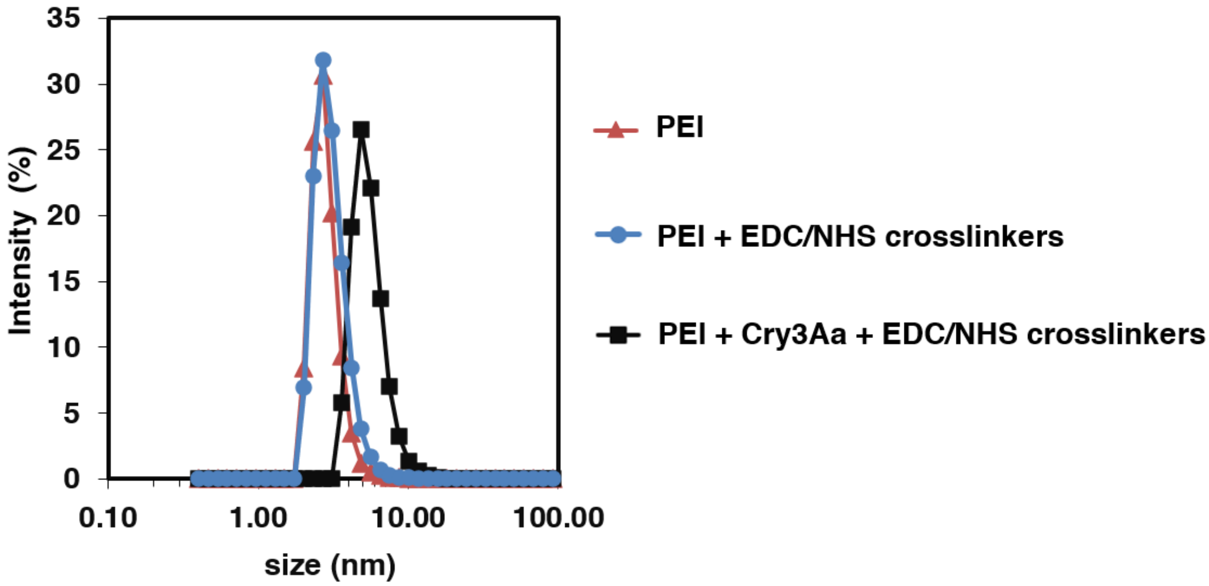

Figure S5. Size distribution of the PEI polymer before and after anchoring to Cry3Aa. PEI exists as a distribution of individual polymers of different sizes with a diameter averaging 5 nm, which coincides with the 5 nm diameter of the Cry3Aa nanochannels. The nanochannel should favor PEI polymers smaller than 5 nm, and thus incubation and crosslinking of PEI to the Cry3Aa crystals should lead to an upward shift in the size distribution. Consistent with this, the average size of the PEI polymer in solution was 5 nm before incubating with PEI-3A crystals and 8 nm afterwards. This indicates that the smaller PEI polymers are the ones that bind to Cry3Aa crystals. This observation is consistent with the binding of PEI within the channels of the Cry3Aa crystal, since the channel diameter would restrict the size of PEI polymer that could enter and thus favor the crosslinking of the smaller PEI polymers, leaving the larger PEI polymers in solution. If the PEI polymer was only reacting on the surface of the Cry3Aa crystals, one would expect the PEI polymer size distribution after Cry3Aa crystal incubation to remain about the same.

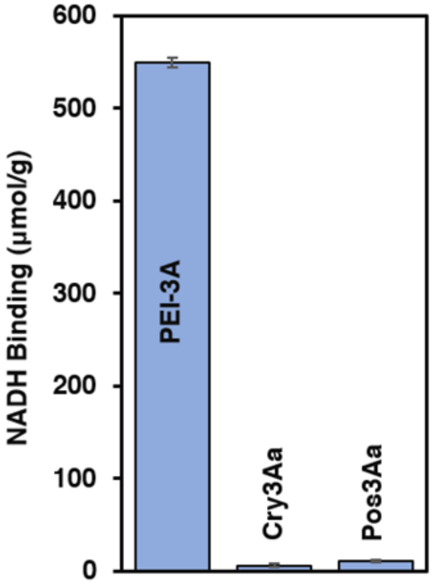

Figure S6. NADH binding capacity of PEI-3A crystals. The cationization of Cry3Aa crystals by PEI polymers increased their NADH binding capacity almost 90-fold and 50-fold in comparison with Cry3Aa and Pos3Aa crystals, respectively.

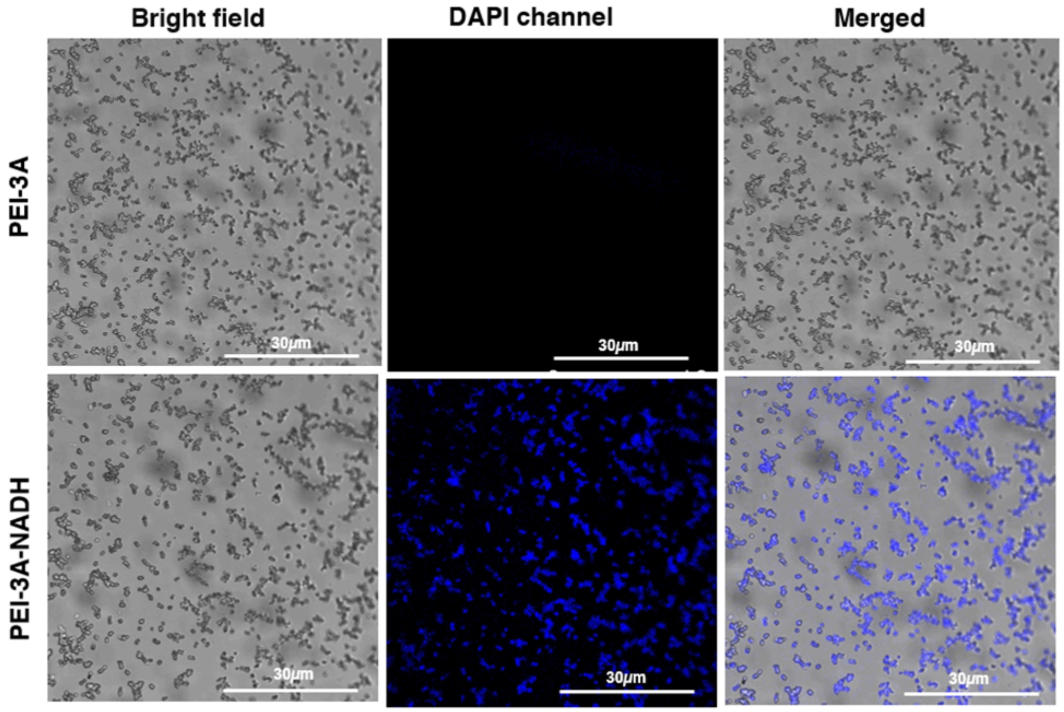

Figure S7. Confocal microcopy of NADH-loaded PEI-3A crystals (PEI-3A-NADH). PEI-3A crystals exhibit no fluorescence signal in the absence of NADH molecules. Upon incubation of PEI-3A crystals with NADH, the resulting crystals exhibited a blue fluorescence, which could be observed using the DAPI channel of the confocal microscope (Leica SP8 confocal microscope).

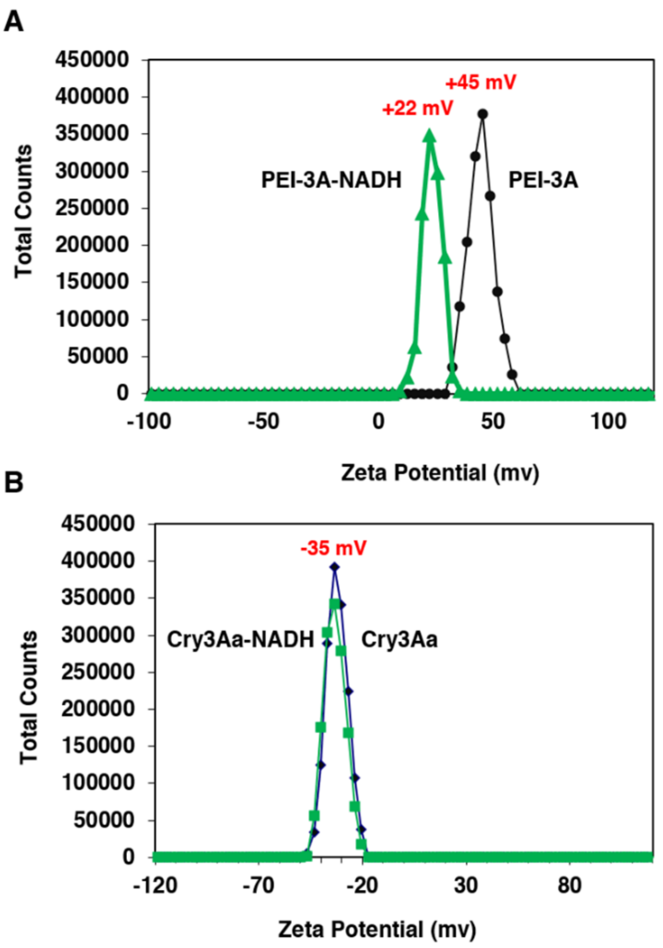

Figure S8. Zeta potential analysis of PEI-3A and Cry3Aa crystals before and after NADH binding. (A) The zeta potential distribution of PEI-3A crystals changed from +45 to +22 when NADH molecules (356 ±4 µmol/g) were bound to them. (B) The zeta potential distribution of Cry3Aa crystals did not exhibit any significant change after NADH binding (4.2 ± 0.2 µmol/g) due to their poor NADH binding capacity.

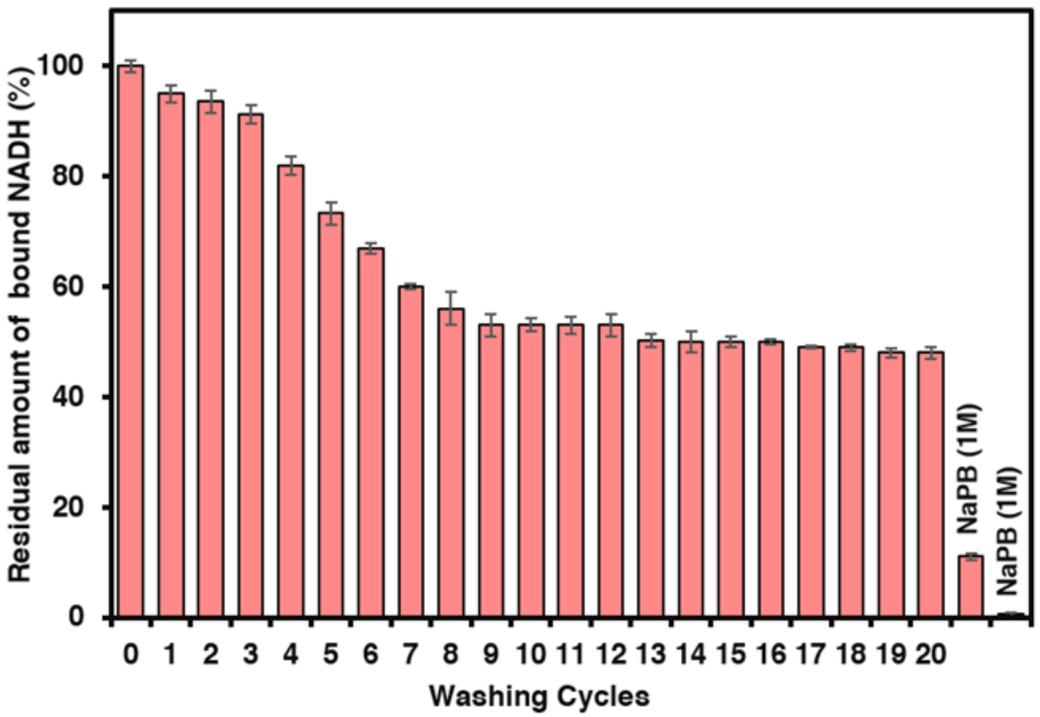

Figure S9. NADH retention by PEI-3A crystals. The retention of NADH bound to PEI-3A crystals over multiple washing cycles with NaPB (10 mM, pH 7.0). During the last cycle, the remaining bound NADH molecules were released from the PEI-3A crystals by washing with 1M NaPB (pH 7.0).

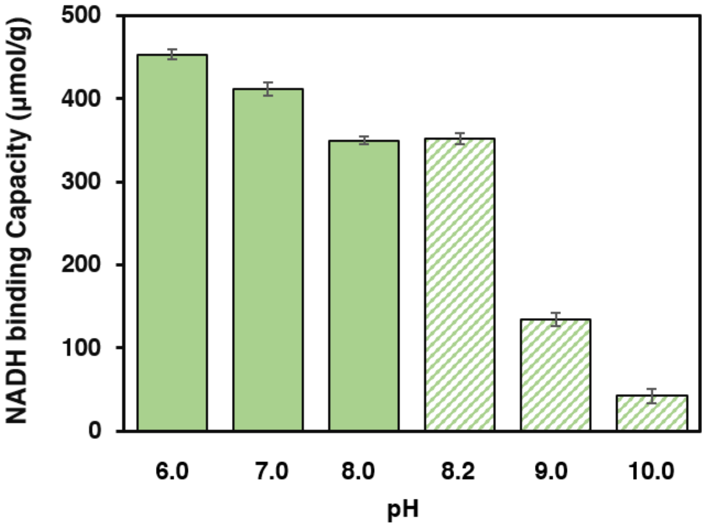

Figure S10. Impact of pH on NADH binding capacity of PEI-3A crystals. The NADH binding capacity of PEI-3A crystals in NaPB (solid bars) (25 mM) and ammonium formate (patterned bars) (25 mM) at different pHs. The deprotonation of amino groups in the bound PEI molecules reduced the NADH binding capacity of PEI-3A crystals as the pH increased.

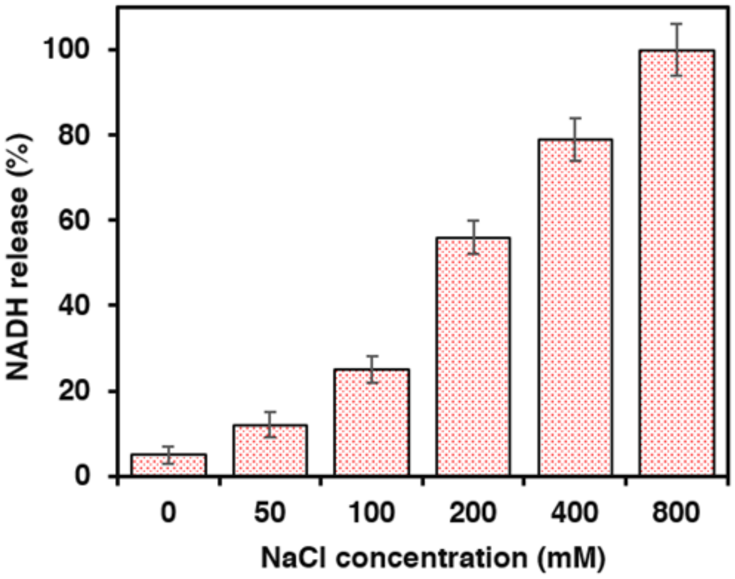

Figure S11. NADH release from PEI-3A crystals as a function of the NaCl concentration.

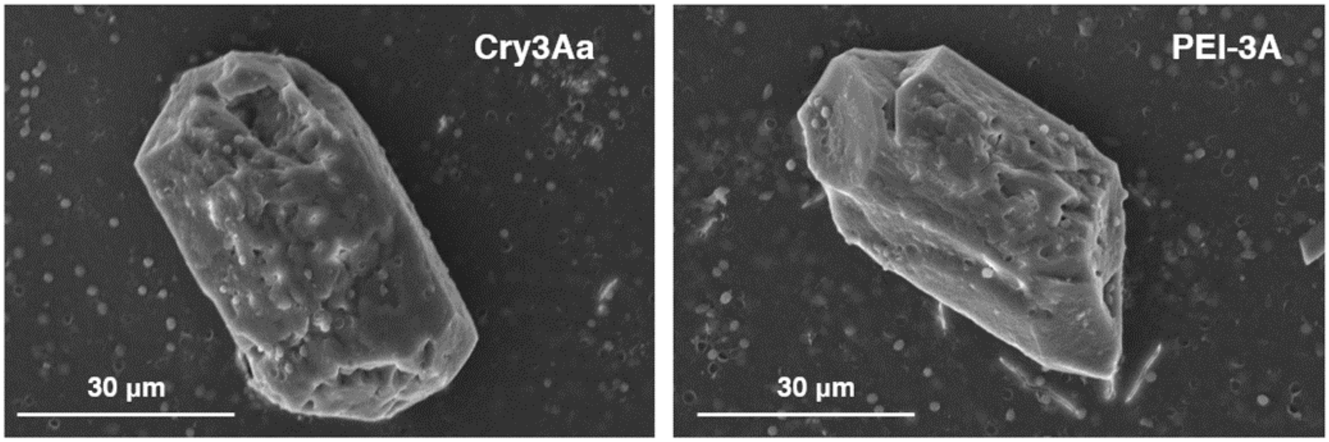

Figure S12. SEM images of *in vitro*-grown crystals. Images of single crystals of (left) Cry3Aa and (right) Cry3Aa functionalized with PEI. The images were collected using a Hitachi SU8000 instrument at 10 kV at 5000× magnification.

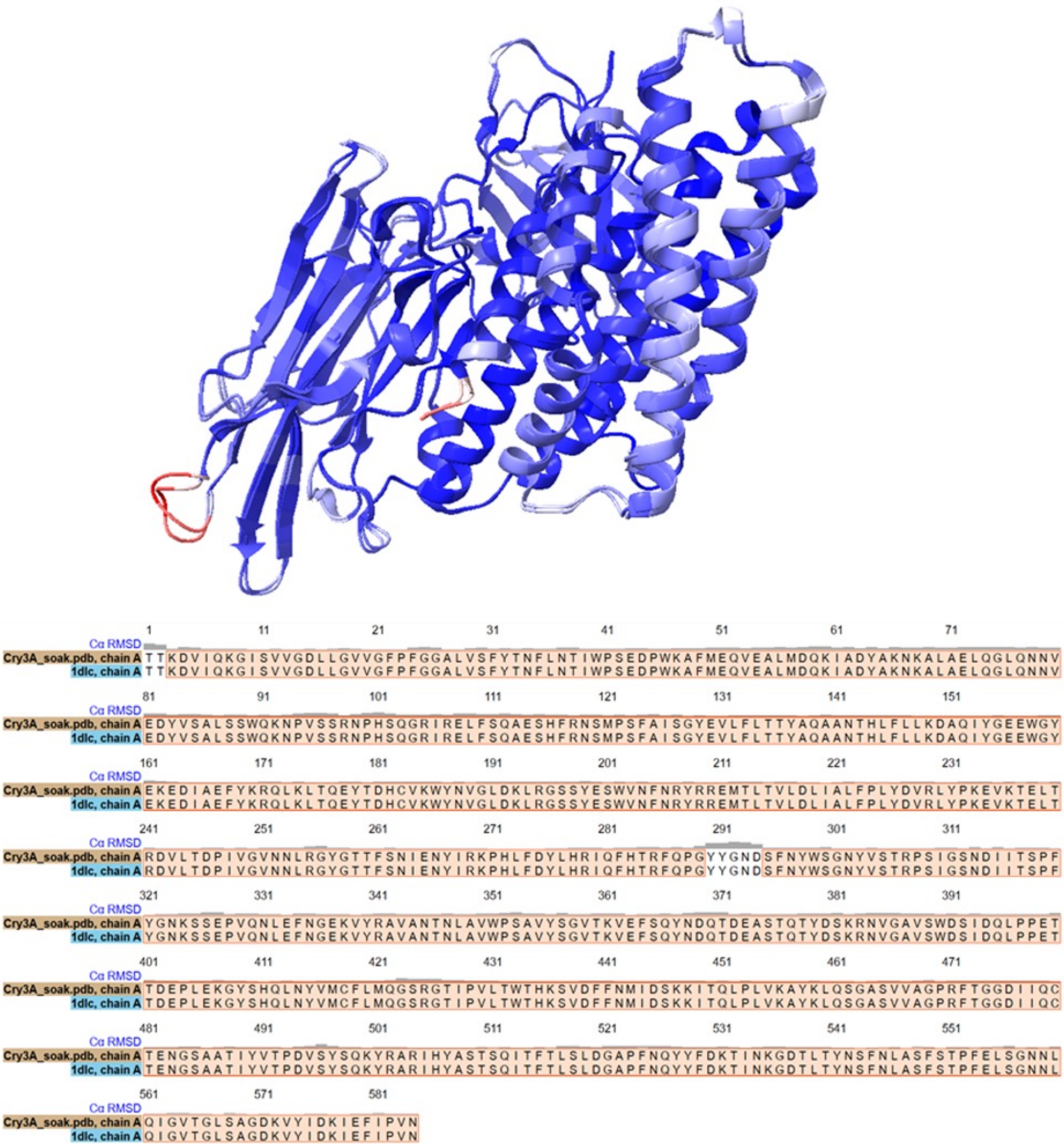

Figure S13. X-ray analysis of PEI-3A crystals. The structure of PEI-3A and the first reported Cry3Aa structure (PDB ID: 1DLC) were superimposed on the RMSD per residue using ChimeraX. RMSD across all 584 pairs: 0.586 Å. The red color highlights regions (290-294 residues) where the deviation is significant, indicating structural variations, flexibility, or differences between the two structures.

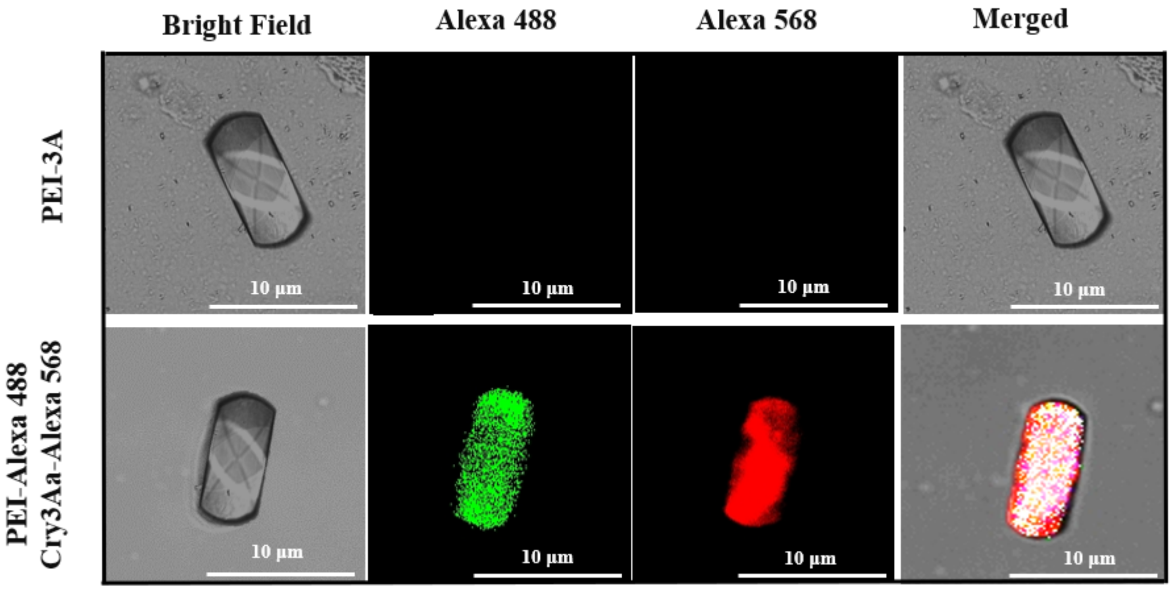

Figure S14. Distribution of PEI molecules within an *in vitro*-grown Cry3Aa crystal. PEI molecules and *in vitro*-grown Cry3Aa crystals were labeled with the green-fluorescent Alexa 488 and the red-fluorescent Alexa 568 fluorophores, respectively. These labeled PEI molecules were then used for the cationization of the tagged Cry3Aa proteins. This crystal is named PEI-Alexa488-Cry3Aa-Alexa568. The data demonstrate that the PEI molecules are distributed throughout the crystal.

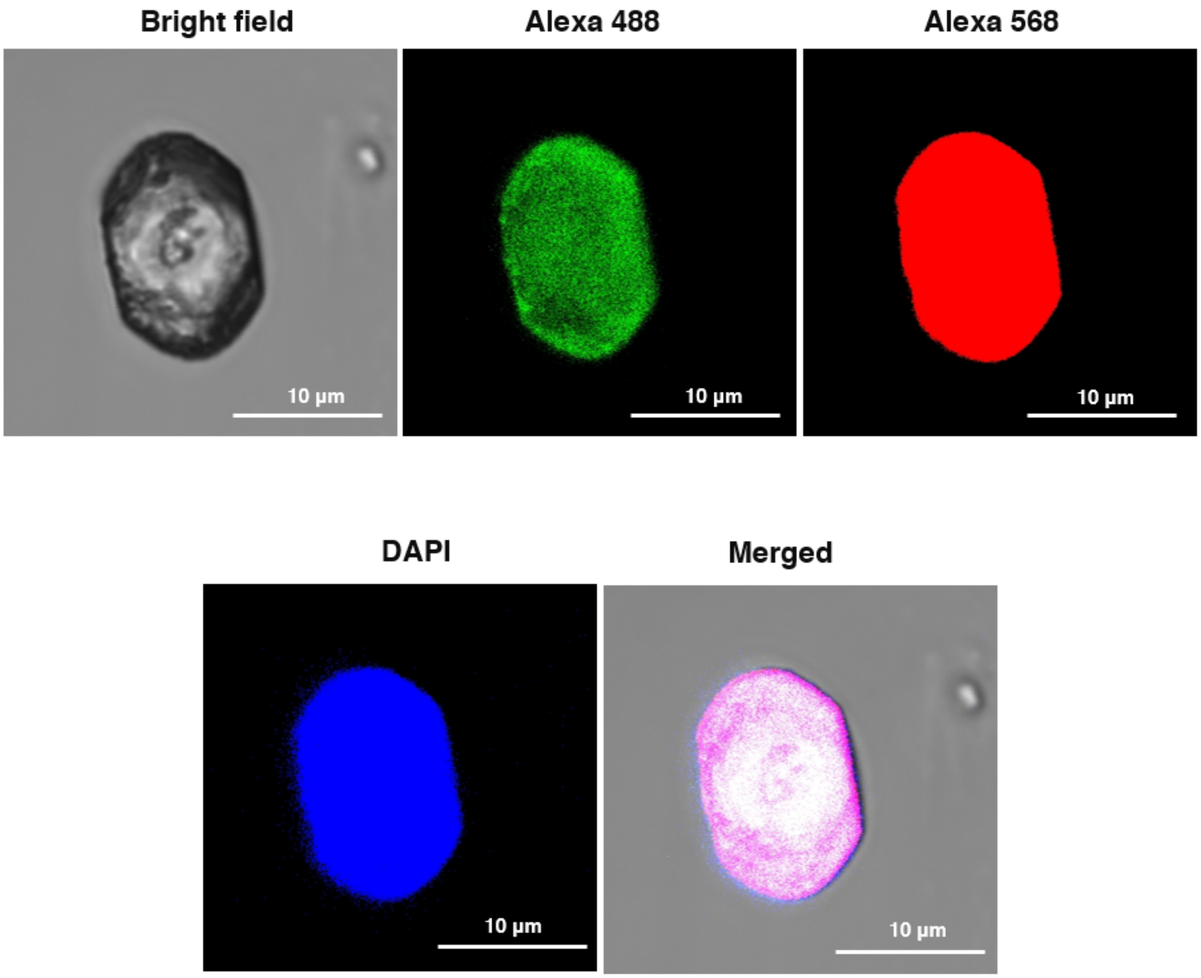

Figure S15. Confocal fluorescence microscopy of NADH-loaded *in vitro*-grown PEI-3A crystals. *In vitro*-grown PEI-Alexa488-Cry3Aa-Alexa568 crystal was mixed with NADH (blue) and imaged by confocal microscopy (Leica SP8 confocal microscope). The results support the distribution of PEI and NADH throughout the channels of the Cry3Aa crystal.

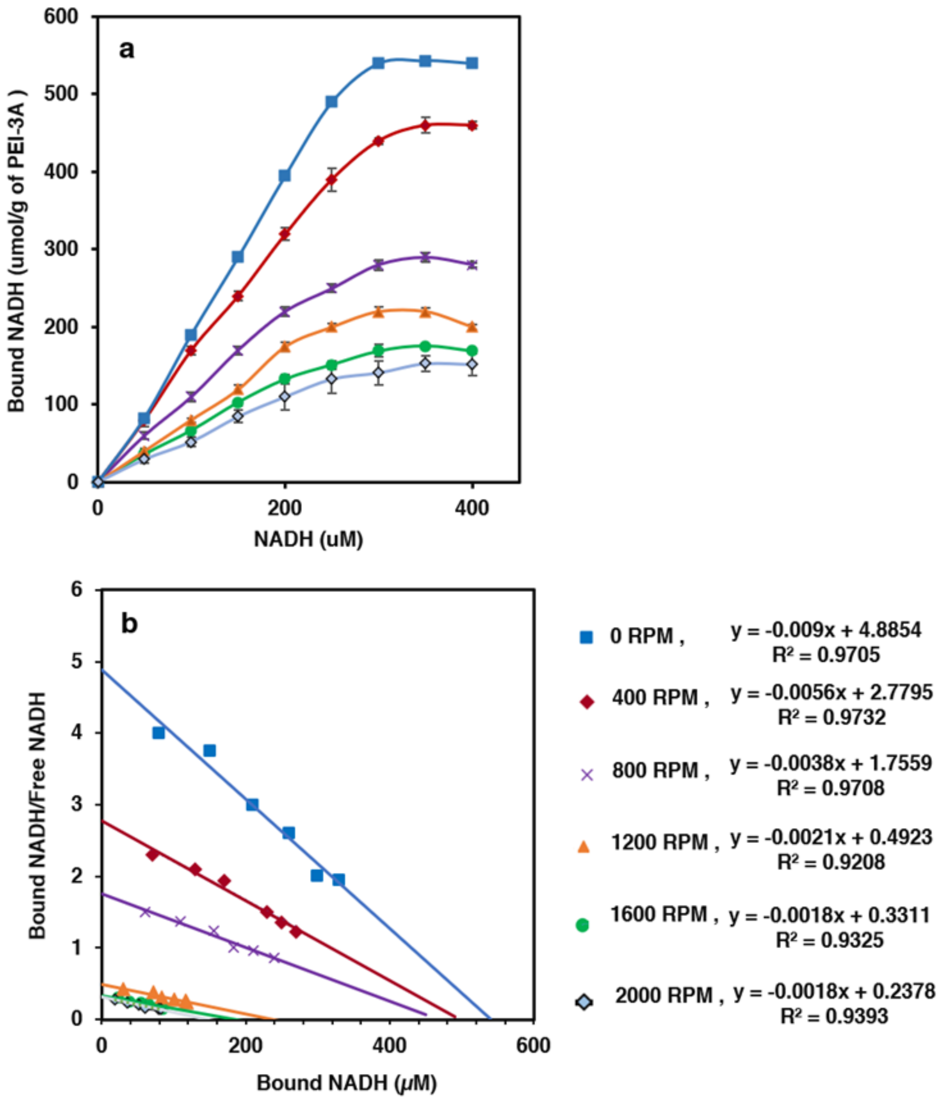

Figure S16. Determination of B_{max} and K_d. (a) Binding isotherms and (b) Scatchard plot for NADH binding to PEI-3A crystals under different orbital shaking speeds (0-2000 RPM) in 10 mM NaPB buffer (pH 7.0). The intercept on the x axis on the Scatchard plot is equal to the B_{max}, while the slope is equal to $-1/K_d$. The determined B_{max} and K_d values are listed in Table S2.

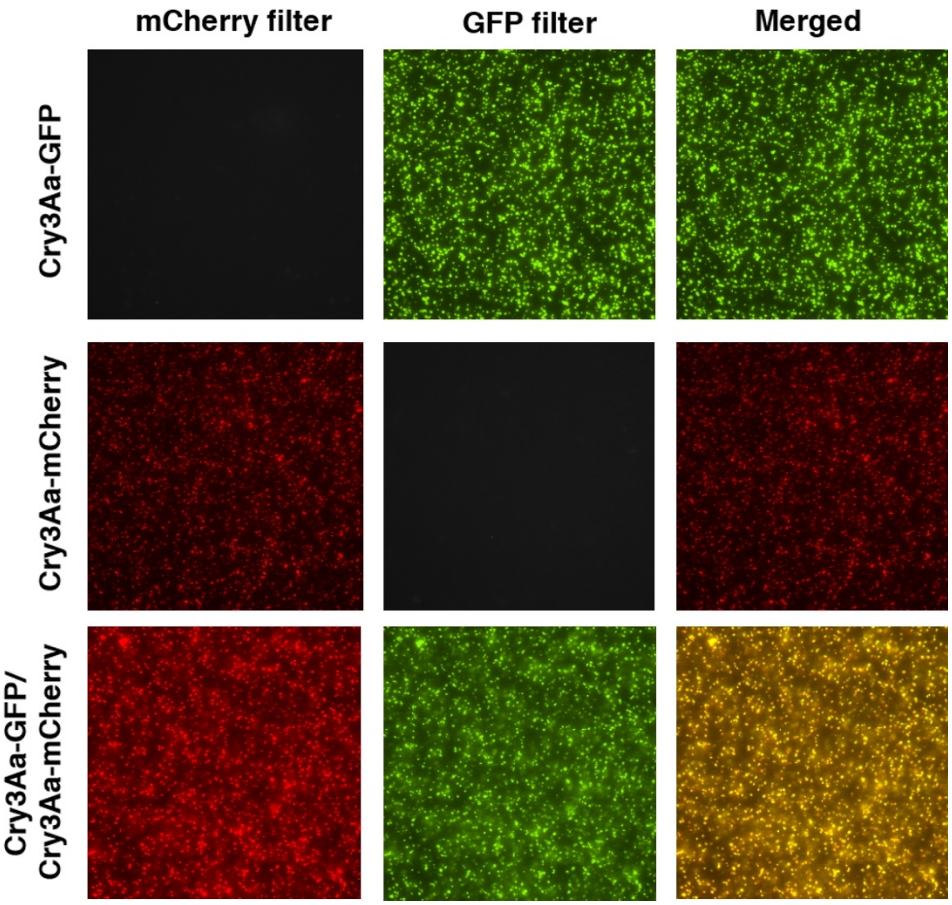

Figure S17. Fluorescence microscopy of particles produced following co-expression of Cry3Aa-GFP and Cry3Aa-mCherry fusion proteins in *Bt*. Particles of Cry3Aa-GFP and Cry3Aa-mCherry fusion proteins co-expressed in *Bt* were imaged by fluorescence microscopy (Nikon TE300 microscope) using the mCherry and GFP filters for determination of the GFP and mCherry localization, respectively. The co-localization of the two reporter proteins is supported by the overlapped fluorescence (yellow) and the Pearson correlation coefficient of 0.956 in the merged image.

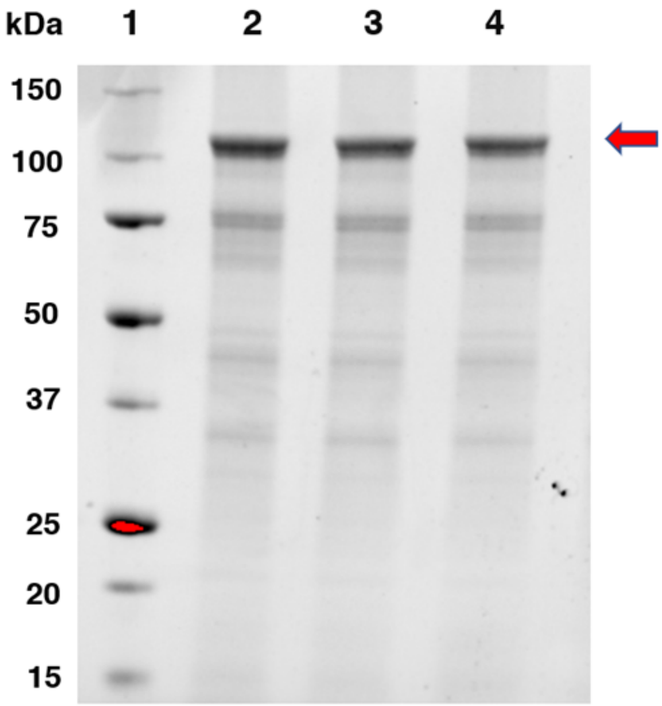

Figure S18. SDS-PAGE analysis of enzyme fusion Cry3Aa particles. The purity of Cry3Aa-FDH, Cry3Aa-LDH, and Cry3Aa-FDH/Cry3Aa-LDH particles was examined using a 10% SDS-PAGE gel after subjecting the proteins to 10 min of boiling. Lane 1: MW Marker; lane 2: Cry3Aa-FDH crystals; lane 3: Cry3Aa-LDH crystals; lane 4: co-immobilized Cry3Aa-FDH/Cry3Aa-LDH crystals. The red arrow points to the band corresponding to Cry3Aa-enzyme fusion bands, which are consistent with their expected molecular weights of 117 kDa for Cry3Aa-FDH and 113 kDa for Cry3Aa-LDH. The co-immobilized Cry3Aa-FDH/Cry3Aa-LDH particles displays a single band for the two Cry3Aa-FDH and Cry3Aa-LDH proteins, presumably because of their similar molecular sizes.

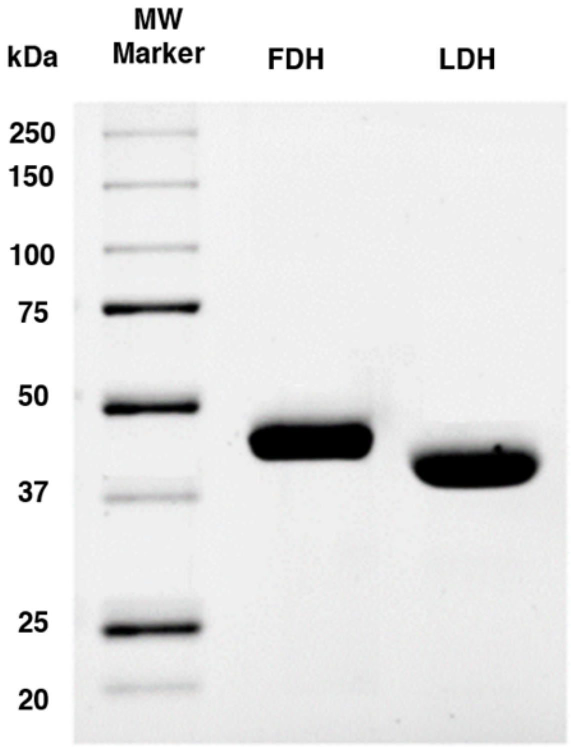

Figure S19. SDS-PAGE analysis of FDH and LDH. The purity of wild-type FDH and LDH proteins was analyzed using a 12% SDS-PAGE gel. The purified FDH and LDH matched the expected molecular weights of 44 kDa and 40 kDa, respectively, based on their respective sequences. The absence of additional bands on the gel confirms their high degree of purity.

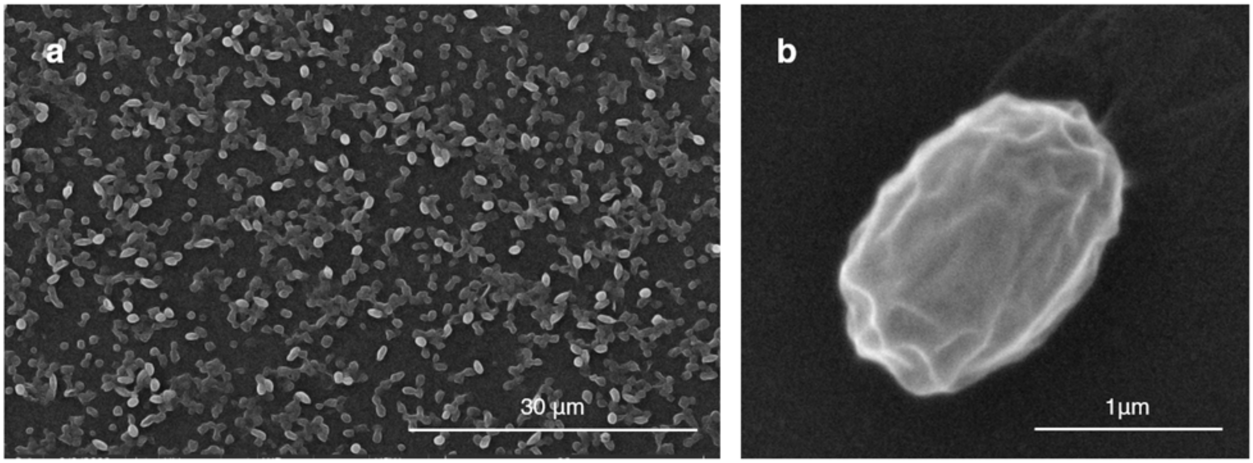

Figure S20. SEM images of Cry3Aa-FDH/Cry3Aa-LDH particles. (a) SEM image of Cry3Aa-FDH/Cry3Aa-LDH particles at 5000× magnification. (b) SEM image of a single Cry3Aa-FDH/Cry3Aa-LDH particle collected at 150000× magnification. Data were collected using a Hitachi SU8000 Scanning Electron Microscope at 30 kV. Based on the images, Cry3Aa-FDH/Cry3Aa-LDH particles are approximately the same size as Cry3Aa crystals, although their forms seem more elliptical.

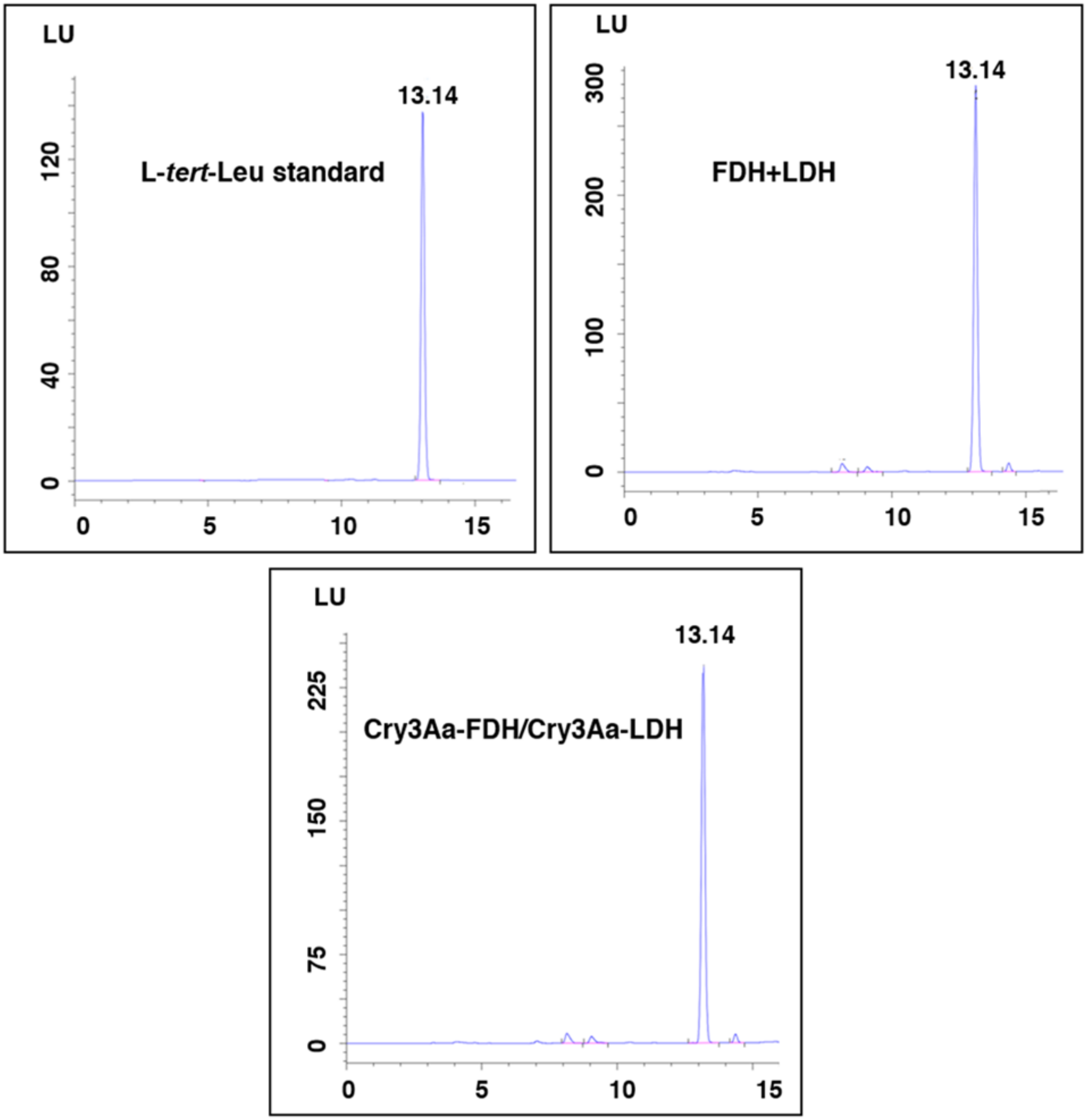

Figure S21. HPLC detection of L-*tert*-Leu modified with OPA. L-*tert*-Leu was modified by OPA and then analyzed by reversed-phased HPLC using a C18 column. The L-*tert*-Leu standard (1 mM) exhibited a retention time peak at 13.14 min, which aligned with the retention time of L-*tert*-Leu produced in the reactions of the FDH+LDH mixture (in a 1:1 molar ratio) and the genetically co-immobilized Cry3Aa-FDH/Cry3Aa-LDH particles.

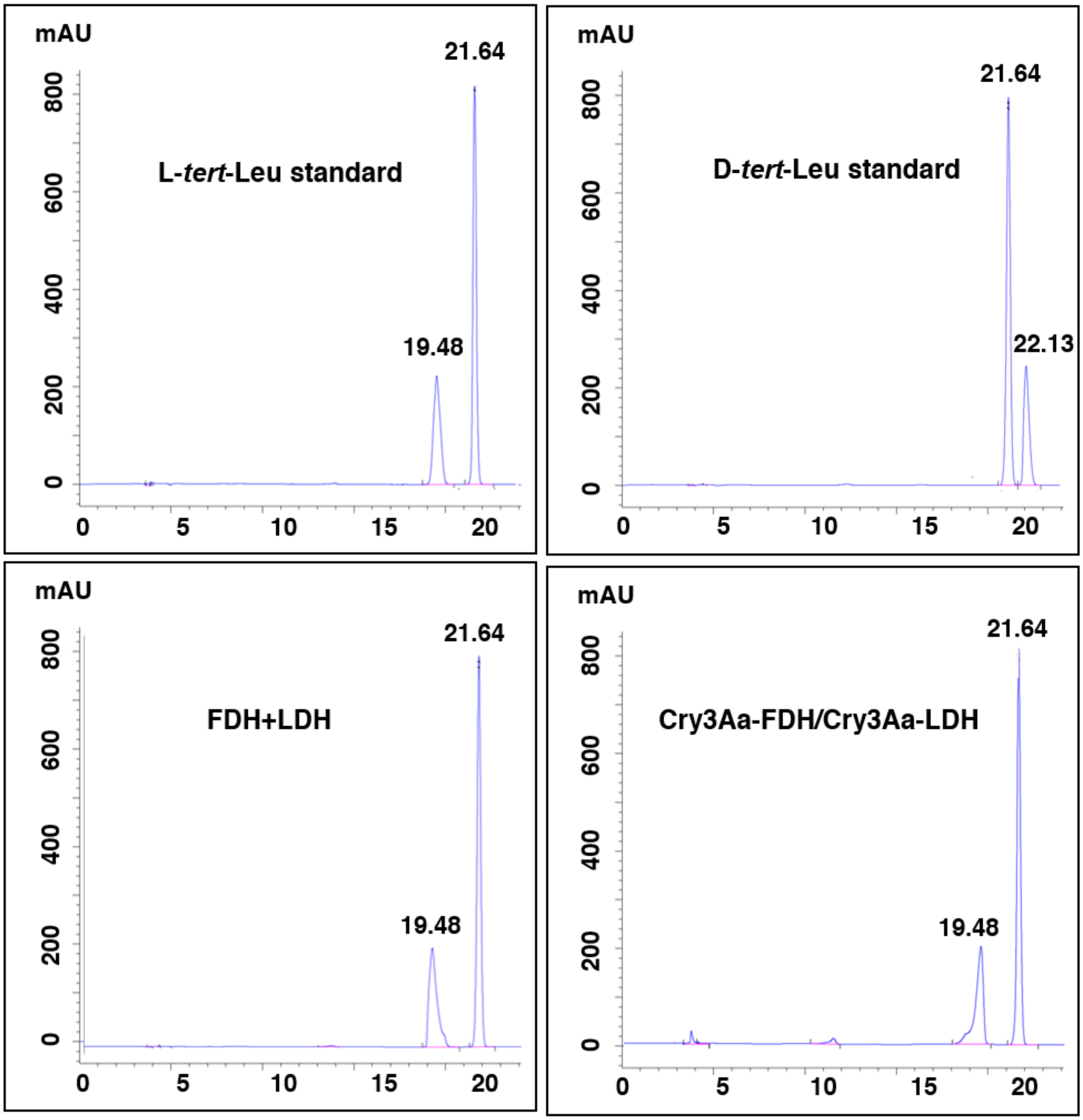

Figure S22. HPLC analysis of the chirality of the *tert*-leu produced by Cry3Aa-LDH enzymes and particles. Marfey's reagent was used to modify the produced *tert*-leu, which was then analyzed by a reverse-phase HPLC system equipped with a C18 column and a UV detector set to 340 nm. Marfey's reagent exhibited a peak at a retention time of 21.64 min. The retention time for L-*tert*-leu indicates a peak at 19.48 min, whereas D-*tert*-leu displays a peak at 22.13 min. It is shown that L-*tert-leu* is predominant (product purity: ≥ 99%) for both FDH+LDH mixture and Cry3Aa-FDH/Cry3Aa-LDH particles.

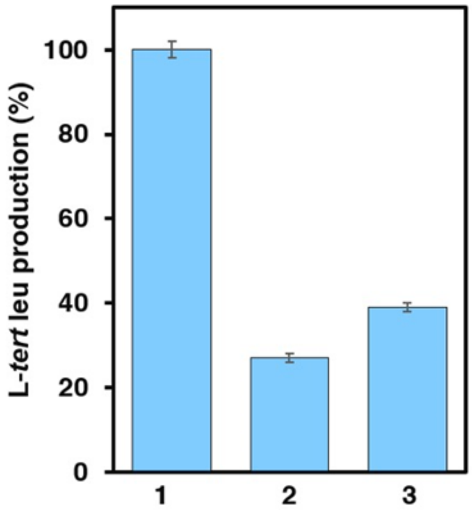

Figure S23. L-*tert*-Leu production efficiency. Lane 1: Production of L-*tert*-Leu by free FDH + LDH (in a 1:1 molar ratio); lane 2: a 1:1 mixture of 3A-FDH and 3A-LDH particles (Cry3Aa-FDH + Cry3Aa-LDH); and lane 3: co-immobilized Cry3A-FDH/Cry3A-LDH particles. 30 µM of each construct was incubated in a reaction buffer containing 50 mM of TMP and 0.5 mM of NADH in ammonia formate (100 mM, pH 8.0) for 24 h at 1000 rpm and 25°C. L-*tert*-Leu produced by free FDH + LDH was considered as 100%. Although the immobilized enzyme systems are less active than the free FDH+LDH enzymes, their advantage lies in their recyclability for multiple reactions. The co-immobilized Cry3Aa-FDH/Cry3Aa-LDH particles exhibit a slightly higher yield than that of the Cry3Aa-FDH+Cry3Aa-LDH mixture, perhaps because of a higher local concentration of the regenerated NADH in the co-immobilized particle.

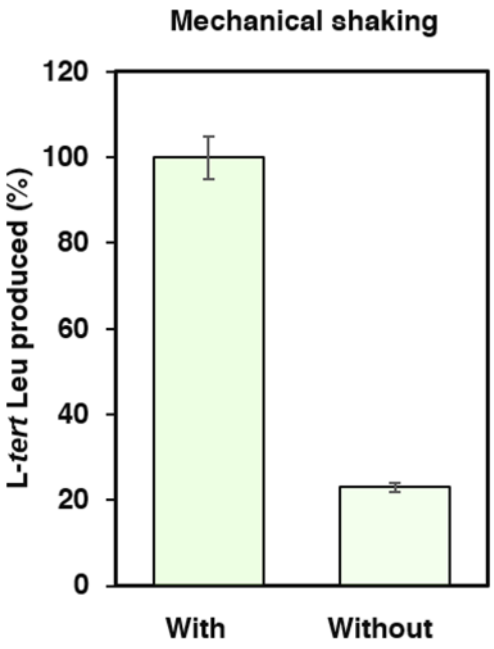

Figure S24. Importance of mechanical shaking for L-*tert*-Leu biosynthesis by Cry3Aa-FDH/Cry3Aa-LDH particles. 106 µM of Cry3Aa-FDH/Cry3Aa-LDH particles were incubated in a reaction buffer containing 25 mM TMP in 30 mM ammonia formate buffer (pH 8.0) for 2 h at 25°C with and without mechanical shaking at 1000 RPM. The samples were then centrifuged at 15000 RPM for 8 min, and the resulting supernatants were analyzed for L-*tert*-Leu production by HPLC. Notably, in the absence of orbital shaking, the L-*tert*-Leu produced was nearly 80% lower. L-*tert*-Leu produced by Cry3Aa-FDH/Cry3Aa-LDH particles under mechanical shaking at 1000 RPM was considered as 100%.

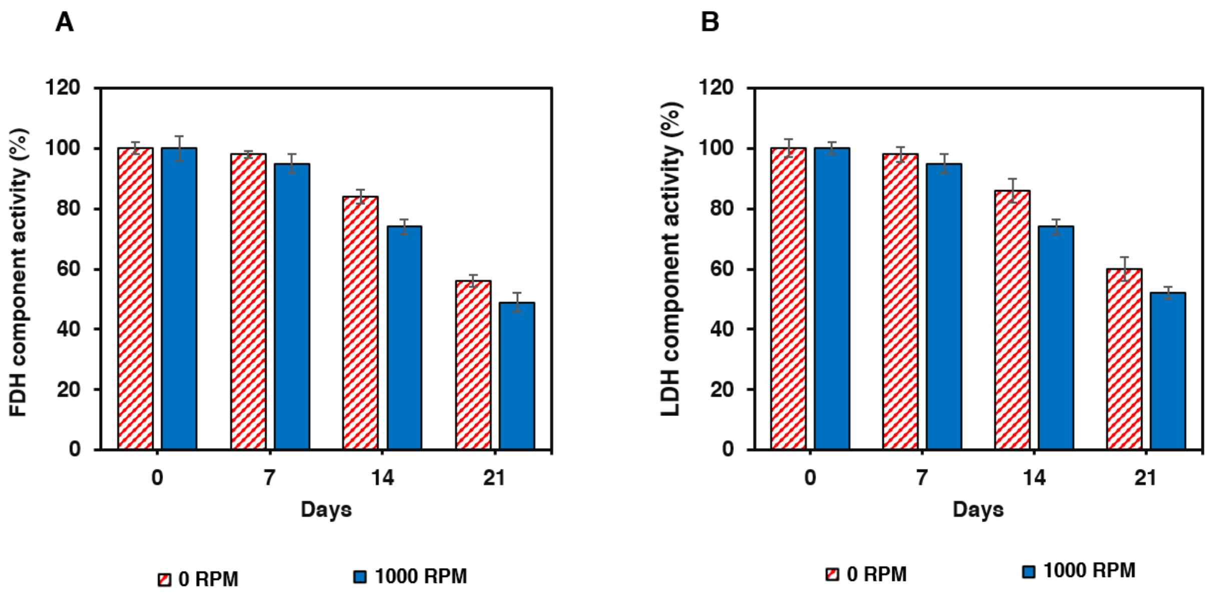

Figure S25. Stability of Cry3Aa-FDH/Cry3Aa-LDH particles under mechanical agitation. (A) Activity of the FDH component in Cry3Aa-FDH/Cry3Aa-LDH particles in the absence or presence of mechanical agitation (0 and 1000 rpm) for over three weeks at 25°C. (B) Catalytic function of the LDH component in the absence or presence of mechanical agitation (0 and 1000 rpm). Both FDH and LDH components exhibited high stability for the first week without losing their catalytic function considerably in the presence or absence of mechanical shaking. However, the activity of both components gradually declined to approximately 50% at 0 and 1000 rpm after three weeks, respectively. Although the particles incubated at 1000 rpm showed only approximately an 8% reduction in activity for both components compared to the particles incubated at 0 rpm, this can be neglected as the mechanical shaking can increase the production yield.

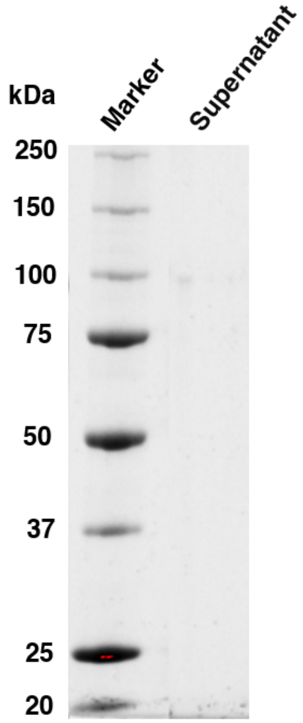

Figure S26. 10% SDS-PAGE of the supernatant of Cry3Aa-FDH/Cry3Aa-LDH particles after mechanical agitation for a 21-day period. No bands were observed on the supernatant, indicating the absence of any protein in the solution. This means that no protein was released from the particles into the solution after three weeks of consistent mechanical shaking.

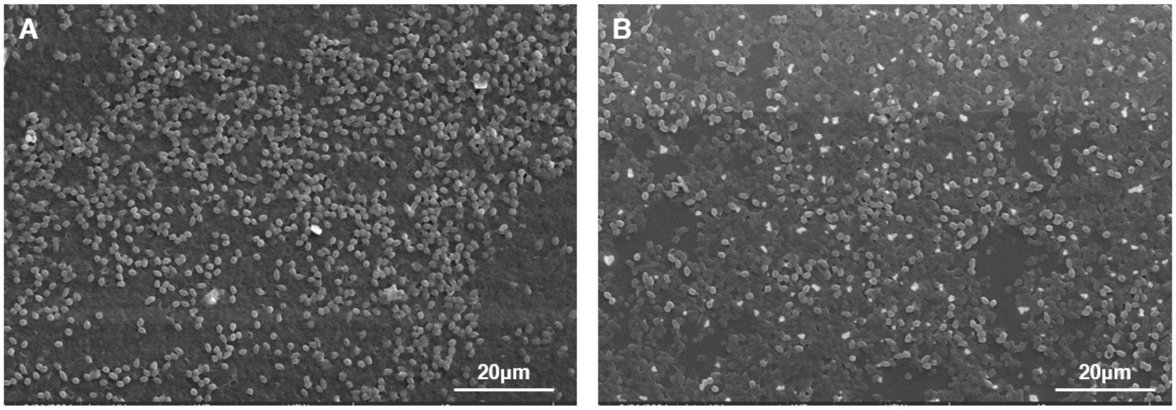

Figure S27. SEM images of Cry3Aa fusion enzyme particles in the absence or presence of mechanical shaking. (A) Global view of Cry3Aa-FDH/Cry3Aa-LDH particles, which were incubated at 0 rpm and 25 °C for 21 days. (B) Cry3Aa-FDH/Cry3Aa-LDH particles were incubated at 1000 rpm and 25 °C for 21 days. The images indicate that the particles retained a similar elliptical shape and size to those incubated without shaking. All images were collected by a Hitachi SU8000 instrument at 20 kV and 5000× magnification.

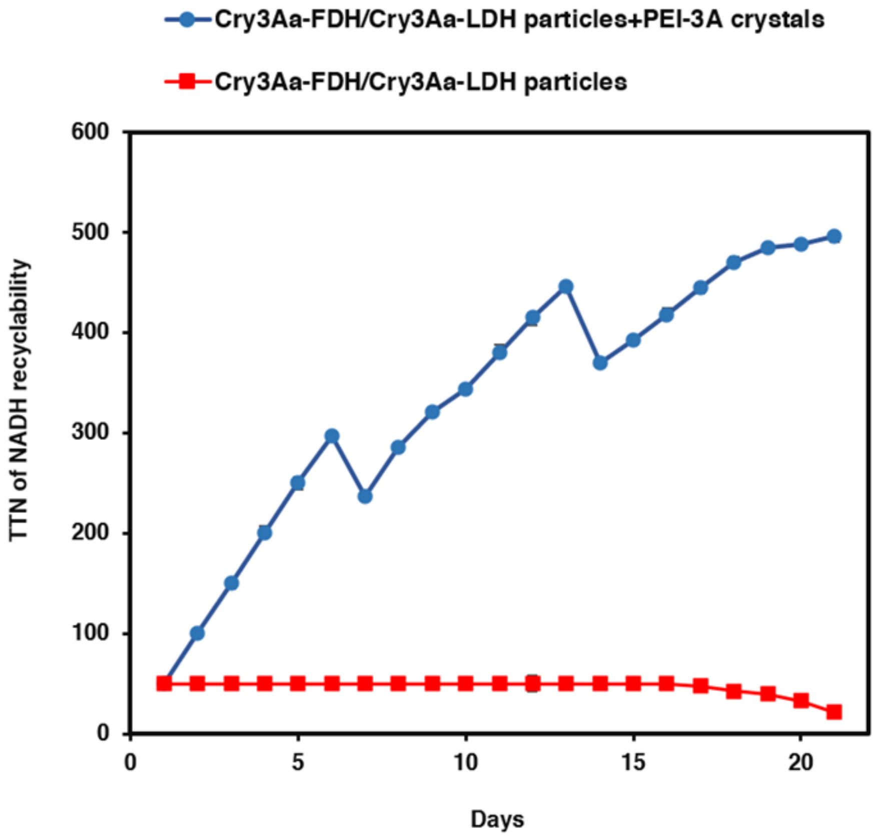

Figure S28. Impact of PEI-3A-mediated NADH recycling on the TTN of NADH. The plot depicts the trend in the net NADH TTN for Cry3Aa-FDH/Cry3Aa-LDH particles with and without PEI-3A-mediated NADH recycling over a 21-day period. In the Cry3Aa-FDH/Cry3Aa-LDH + PEI-3A system, the TTN of NADH gradually increased, reaching close to 500 due to product accumulation and reduced consumption of NADH molecules due to recycling. In contrast, Cry3Aa-FDH/Cry3Aa-LDH particles alone (i.e. without PEI-3A-mediated recycling) had a steady TTN of 50 for the initial 15 cycles due to the required cofactor addition each cycle. The drop in the NADH TTN in the later cycles is likely due to the deterioration of either LDH, FDH, or both enzymes over time.

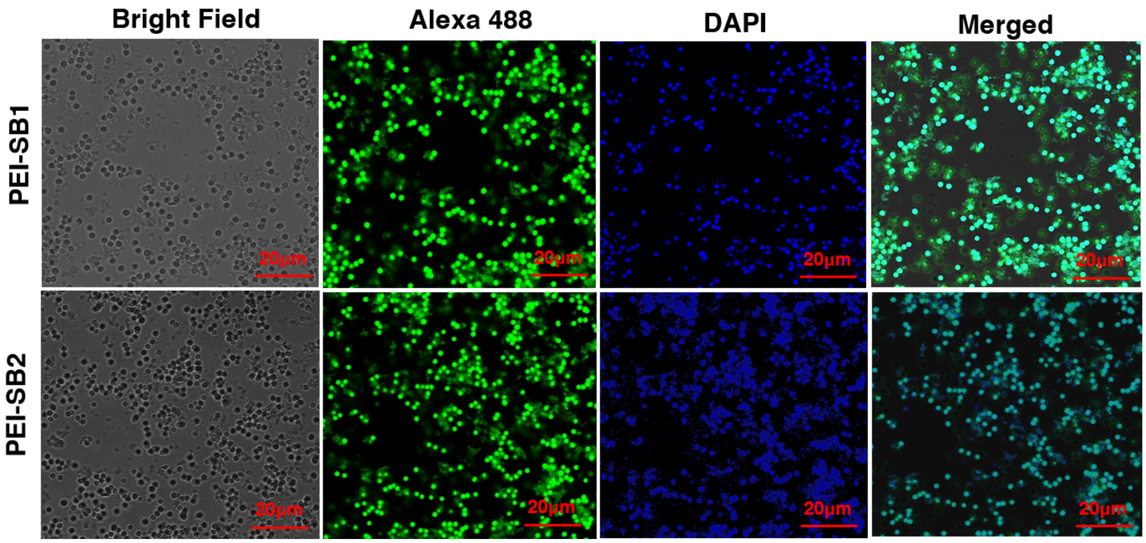

Figure S29. PEI modification of silica beads and subsequent NADH binding. Modification of micrometer-sized silica beads with PEI-labelled-Alexa 488, and their subsequent binding to NADH. PEI-Alexa 488 was successfully anchored to silica beads, as confirmed by their fluorescence in the Alexa 488 channel. The blue fluorescence in the DAPI channel is indicative of NADH binding to the PEI-modified silica beads. The co-immobilization of PEI and NADH is evidenced by the overlap of their fluorescence in the merged image. PEI-modified silica beads with different bead and pore sizes are shown as PEI-SB1 (1 µm beads with 4 nm pores) and PEI-SB2 (1 µm beads with 10 nm pores).

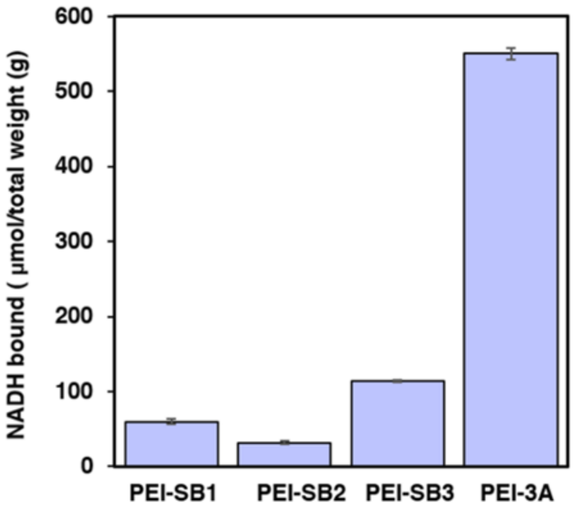

Figure S30. Comparison of NADH binding capacity between PEI-modified silica beads and PEI-3A crystals. Binding capacity of NADH to PEI-SB1, PEI-SB2, and PEI-SB3 beads, and PEI-3A crystals. For the same amount of anchored PEI, PEI-3A crystals exhibit an almost 5-fold higher NADH binding capacity relative to PEI-SB.

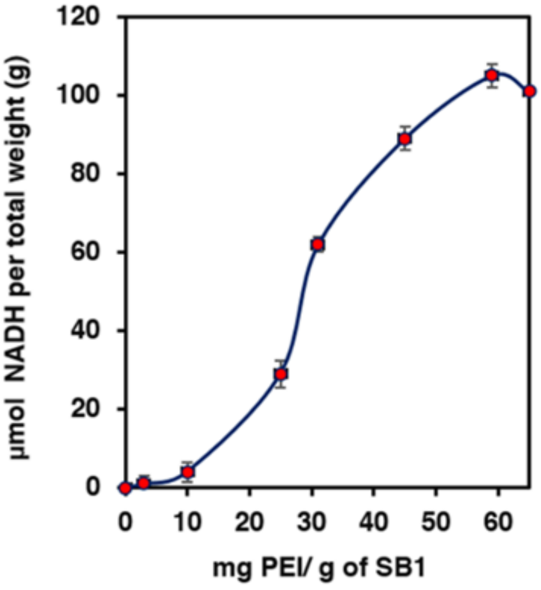

Figure S31. Relationship between the amount of PEI anchored to SB1 and its NADH capacity.

Supporting Tables

Table S1. Data collection and refinement statistics of PEI-3A crystal.

	**PEI-3A**
**Data collection**	
Wavelength (Å)	0.99984
Space group	C 2 2 21
Cell dimensions	
a, *b*, *c* (Å)	115.846, 133.368, 105.798
Resolution (Å)	20 (3.1)
R_{merge}	0.194 (0.836)
$I / \sigma I$	13.3 (2.25)
Completeness (%)	99.9 (100.0)
Redundancy	13.0 (13.3)
**Refinement**	
Resolution (Å)	3.2
No. reflections	14992
R_{work} / R_{free}	19.26/ 27.54
No. atoms	
Protein	4697
Ligand/ion	0
Water	4

Outer shell statistics are in parentheses.

Table S2. NADH binding affinity to PEI-3A crystals at different orbital shaking speeds. The results indicate that raising the orbital shaking speed from 0 to 2000 rpm leads to a nearly 3-fold decrease in B_{max} value and a 5-fold increase in K_d.

**RPM**	**K_d (µM)**	**B_{max} (nmol/mg)**
0	111 ± 1	540 ± 6
400	178 ± 1	495 ± 2
800	263 ± 1	445 ± 3
1,600	555 ± 5	180 ± 4
2,000	565 ± 6	152 ± 5

Table S3. Kinetic parameters of FDH, LDH, and genetically co-immobilized Cry3Aa-FDH/Cry3Aa-LDH particles.

**Enzymes**	**K_m (mM)**	**V_m (µmol/min)**	**Specific Activity (U/mg)**
**FDH**	15.3 ± 0.3	29.4 ± 0.6	9.6 ± 0.2
Cry3Aa-**FDH**/Cry3Aa-LDH[a]	210.2 ± 0.2	25.6 ± 0.3	1.6 ± 0.1
**LDH**	23.8 ± 0.1	23.9 ± 0.1	7.96 ± 0.1
Cry3Aa-FDH/Cry3Aa-**LDH**[a]	256.4 ± 0.2	20.1 ± 0.2	1.25 ± 0.2

[a] For the co-immobilized crystals, the bolded enzyme is the one whose activity is being evaluated. The specific activities of FDH and LDH decreased approximately 6-fold when genetically fused to Cry3Aa crystals.

Table S4. The density of PEI-3A crystal and silica beads. This table shows that PEI-3A is approximately 60% less dense than silica beads.

Silica bead type	PEI-SB1	PEI-SB2	PEI-SB3	PEI-3A
**Density (g/cm^3)**	1.47 ± 0.9	1.49 ± 0.3	1.21 ± 0.5	0.61 ± 0.4
**Size (μm)**	1	1	0.15	~1
**Pore size (nm)**	4	10	4	5

Table S5. List of primers utilized for cloning (5' → 3'). Restriction sites are underlined.

pET28- LDH Fwd	CCGCGCGGCAGCCATATGACATTAGAAATCTTCG
pET28- LDH Rev	GTGGTGGTGGTGGTGCTCGAGTTAGCGACGGCTAATAATATCG
pHT315-Cry3Aa-FDH Fwd	GAATTTATTCCAGTGAATGGATCCGCAGCTGCGATGGCTAAAATACTGTGTGTCCTG
pHT315-Cry3Aa-FDH Rev	CAGTGAATTCGAGCTCGGTACC TCATCCGGCTTTCTTGAACTTAGC
pHT315-Cry3Aa-LDH Fwd	TTTATTCCAGTGAATGGATCCGCAGCTGCGATGACATTAGAAATCTTCG
pHT315-Cry3Aa-LDH Rev	CAGTGAATTCGAGCTCGGTACCTTAGCGACGGCTAATAATATCGTGACCG
pHT315-Cry3Aa-FDH-rbs-Cry3Aa-LDH Fwd	AAGAAAGCCGGATGAGGTACCAAAGGGAGGAAGAAAAATGAATCCGAACAATCGAAGTG
pHT315-Cry3A-FDH-rbs-Cry3A-LDH Rev	CAGTGAATTCGAGCTCGGTACCTTAGCGACGGCTAATAATATCGTGACCG

Calculations

PEI vs Cry3Aa ratio

The average volume of the crystal was calculated to be 1.6 μm × 0.80 μm × 0.80 μm or 1.1×10^9 nm^3 by measuring the volume of ~100 Cry3Aa crystals.[2] By analyzing the crystal lattice dimensions (117 Å × 134 Å × 104 Å), the volume of the unit cell can be determined to be 1.6×10^6 Å^3. Additionally, the crystallographic data indicates that there are 8 molecules of Cry3Aa within each unit cell. Then, the following formula was used to calculate the number of Cry3Aa molecules in a single Cry3Aa crystal:

$$\text{Molecules of Cry3Aa per crystal} = \frac{\text{Vol. of Cry3Aa crystal}}{\text{Vol. of Unit Cell}} \times \text{ Cry3Aa molecules per unit cell}$$

$$\text{Molecules of Cry3Aa per crystal} = \frac{1.1 \times 10^9\ \text{nm}^3}{1.6 \times 10^3 \text{nm}^3} \times\ 8 = 5.5 \times 10^6$$

This calculation indicates that a single Cry3Aa crystal has approximately 5.5×10^6 Cry3Aa molecules per crystal. Considering the molecular weight of Cry3Aa (73 kDa), the average weight of a single Cry3Aa crystal can be estimated by the following formula:

$$\text{Weight of a single Cry3Aa crystal} = \text{Cry3Aa molecules per crystal} \times \text{MW of Cry3Aa}$$

One Dalton (Da) is equal to 1.6×10^{-24} g, therefore, one molecule of Cry3Aa is approximately 1.2×10^{-19} g. Therefore, we have:

$$\text{Weight of a single Cry3Aa crystal} = (5.5 \times 10^6) \times 73\ \ \text{kDa}$$

$$= 6.4 \times 10^{-13}\text{g}$$

The molar ratio of PEI and Cry3Aa molecules can be calculated using the data obtained using the Cu(II)-assay (12 ± 0.3 mg of PEI per g of Cry3Aa crystals). Based on the size distribution analysis of PEI, it was shown that smaller molecules have a higher tendency to bind and penetrate Cry3Aa crystals. To determine the molarity of PEI that can bind to 1 g of Cry3Aa crystals, the Mn value, the average statistical weight based on the number of molecules with that weight, for PEI (60 kDa), was used for the calculation. Considering the weight of a single Cry3Aa crystal (6.4×10^{-13} g), the number of PEI molecules that can bind per single Cry3Aa crystal can be calculated by using Avogadro's number (6.022×10^{23}) as $\sim 8 \times 10^{4}$.

Supporting Methods and Materials

Materials

3-aminopropyl-triethoxysilane (APTMS), O-phthalaldehyde (OPA), 1-fluoro-2,4-dinitrophenyl-5-L-alanine amide, nicotinamide adenine dinucleotide (NAD(H)), trimethylpyruvic acid (TMP), L-*tert*-leucine, and D-*tert*-leucine were obtained from the TCI company. Ammonia formate, polyethylenimine (PEI) (Mn: 60 kDa and Mw: 750 kDa), and other buffer salts were also purchased from Sigma-Aldrich. Additional compounds and chemicals are given in parenthesis.

Experimental Section

Production of PEI-3A crystals

Cry3Aa crystals were produced by transforming *Bacillus thuringiensis* (*Bt 407G*) cells with a previously prepared PHT-Cry3Aa plasmid and growing the cells for three days as described previously.[3] The cells were harvested and lysed with lysozyme, and the Cry3Aa crystals were purified via low-speed centrifugation (6000 rpm) after washing with 10% hexane to remove the spores.[4] A solution of Cry3Aa in ddH_2O was prepared at a concentration of 5 mg/mL based on Bradford. To measure the amount of Cry3Aa in solution more accurately, 2 mL of this solution was aliquoted and dehydrated, and then the weight of the dehydrated Cry3Aa crystals was determined. Based on this value, a volume containing 25 mg of Cry3Aa crystals was aliquoted and centrifuged at 8500 rpm, after which the supernatant was removed. The resulting pellet was resuspended in 50 mL of 50 μg/mL PEI solution (pH 7.0), and then EDC/NHS (1:2 molar ratio) was gradually added with magnetic stirring to promote the anchoring of PEI to the carboxylates within

the nanochannels and surface of the Cry3Aa crystals. After 5 h, the reaction was quenched using a solution of 100 mM Tris buffer at pH 7.0. Subsequently, the samples were centrifuged, and the supernatant was retained for subsequent PEI analysis. The pellet was then washed multiple times with NaCl (100 mM), followed by ddH_2O to remove any weakly bound PEI molecules.

PEI reacts with Cu(II) sulfate to produce a blue complex. The amount of PEI bound in the PEI-Cry3Aa crystals was calculated by determining the amount of PEI in solution before and after the reaction with the Cry3Aa crystals. Following binding of PEI to the Cry3Aa, the resulting crystals were separated by centrifugation, and the resulting supernatant was treated with 130 mM of copper(II) sulphate and then the absorbance at 285 nm was measured. Then, a standard curve was generated by measuring the A_{285} for a series of solutions containing a fixed amount of 130 mM of Cu(II) sulfate in the presence of different amounts of PEI (0-50 µg/mL) at pH 7.0.[5, 6] The amount of PEI remaining in solution could then be determined based on A_{285} and the calibration curve. The amount of PEI bound to Cry3Aa crystals could be determined based on the difference between the initial amount of PEI provided and the amount of PEI remaining in solution.

Dynamic light scattering (DLS) of Cry3Aa and PEI-3A crystals

The zeta potential and hydrodynamic diameter of Cry3Aa and PEI-3A crystals were measured at 25°C using a Malvern Zetasizer Nano ZS90 (Malvern Instruments, UK). 100 µg/mL crystals were individually prepared in ddH_2O solution for measurement of size distribution and PBS (10 mM) for zeta potential analysis.

Binding of NADH to PEI-3A crystals

The binding capacity of NADH to PEI-3A crystals was determined by incubating PEI-3A crystals (50 µg/mL) with NADH (0.25 mM) at 10°C for 30 min under gentle rotation. Then, the amount of NADH bound to the PEI-3A crystals was quantified by measuring the absorbance of the supernatant at a wavelength of 340 nm using an Eppendorf BioSpectrometer (Eppendorf AG, Germany) after centrifugation at 15,000 rpm for 5 min. The NADH binding into the crystals was then defined as the number of moles of NADH bound per unit of dried weight of PEI-3A crystals, which are shown in the PEI-3A-NADH crystals (dried weight of PEI-3A, moles of bound NADH). The binding of NADH molecules was further studied by confocal microcopy using a Leica SP8 confocal microscope in the DAPI channel. The excitation and emission wavelengths for NADH fluorescence were tuned to 335-350 nm and 440-470 nm, respectively.[7] To compare the binding capacity of PEI-3A crystals with Cry3Aa and Pos3Aa crystals, the same experiment was conducted for these unmodified crystals.

Zeta Potential Measurement of PEI-3A and Cry3Aa crystals before and after NADH binding

The zeta potential of PEI-3A and Cry3Aa crystals before and after binding NADH molecules was individually measured at 25°C using a Malvern Zetasizer Nano ZS90 (Malvern Instruments, UK). For this aim, 0.25 mg/mL of the crystals were individually incubated in the absence or presence of NADH (0.5 mM) in ddH_2O solution for 10 min under slow rotation. The samples were then centrifuged at 15000 rpm for 5 min and

washed multiple times with ddH_2O to remove all unbound NADH molecules. 100 µg/mL of the crystals were then provided in PBS (10 mM, pH 7.0) for zeta potential analysis.

In vitro **growth of Cry3Aa crystals**

Cry3Aa protein crystals were dissolved in a solution containing 50 mM Na_2CO_3, 150 mM NaCl, and adjusted to pH 11. To purify the Cry3Aa protein further, gel filtration (Bio-rad ENrichTM SEC 650) was employed. *In vitro*-grown Cry3Aa crystals were produced by the sitting-drop vapor diffusion method. This involved combining 1 µL of protein solution (8.3 mg/mL) with 1 µL of the well solution (20% (v/v) 1, 4-butanediol, 100 mM HEPES/NaOH pH 7.5, 200 mM NaCl) in a 96-well crystallization plate (Hampton) incubated at 10°C.[8] The Cry3Aa crystals obtained were gently transferred to a solution containing 100 mM HEPES/PEI (50 µg/mL) and EDC/NHS coupling reagents (5 mM and 10 mM, respectively) at pH 7.5. The samples were incubated for 2 h at room temperature, followed by treatment with 100 mM Tris buffer (pH 7.5) for 30 min to deactivate any residual EDC/NHS cross-linkers. Subsequently, the samples were centrifuged and washed multiple times with HEPES (100 mM) and then ddH_2O to remove excess salts before being stored at 4°C for further use.

Crystallization, data collection, phase determination and refinement

PEI-3A crystals were incubated in a mother liquor solution containing 30% glycerol cryoprotectant, and then flash-cooled in liquid nitrogen. X-ray diffraction datasets were subsequently collected on the microfocus beamline 05A at NSRRC, utilizing the RAYONIX MX-300 HS detector. Data processing and scaling were performed using the HKL2000 software.[9] To generate the initial structure, PHASER was employed under the

CCP4i interface, with the Cry3Aa structure (PDB ID:1DLC) serving as the search model.[10] Iterative cycles of model building, and refinement were conducted using the Coot, Refmac5, and Phenix programs to enhance the quality of the model.[11-14]

Scanning electron microscope (SEM) of PEI-3A crystals

Scanning electron microscopy (SEM) was used to study the morphology of *in vitro* and *in vivo*-grown Cry3Aa and PEI-3A crystals. After incubating the samples in ddH_2O (0.2 mg/mL), 5 µL was placed on a carbon glass and allowed to dry gradually overnight before being coated with 80/20 Pd/Pt using a Q150T ES turbomolecular pumped coater (Quorum). The images were captured using a Hitachi SU8000 instrument.

Distribution of PEI and NADH molecules within *in vivo*-grown PEI-3A crystals

The localization of PEI polymer within Cry3Aa crystals was assessed by first labeling the *in vitro*-grown crystals by mixing them with Alexa Fluor™ 568 C5 Maleimide (Thermo Fisher Scientific) (1 mM) in 100 mM HEPES buffer (pH 7.5) for 1 h under gentle rotation. Subsequently, the crystals were thoroughly washed multiple times with the same buffer to remove any unbound Alexa 568 molecules. In parallel, a solution of PEI (50 µg/mL) was incubated with Alexa Fluor™ 488 NHS Ester (N-hydroxysuccinimide ester) (Thermo Fisher Scientific) (1 mM) in 100 mM HEPES buffer (pH 7.5) for 1 h under gentle rotation. Following this, the PEI solution was washed with 100 mM HEPES buffer using a centrifugal filter (3 kDa, Amicon® Ultra) multiple times to obtain the labeled PEI-Alexa488 polymer. Finally, the labeled Cry3Aa-Alexa 568 and PEI-Alexa 488 polymers were utilized for the cationization procedure to produce Cry3Aa-Alexa 568-PEI-Alexa 488 crystals. The obtained crystals were then incubated in the presence of NADH (0.25 mM) in ddH_2O

under slow rotation at 10°C for 30 min. After several ddH_2O washings to remove the unbound NADH molecules, the Cry3Aa-Alexa 568-PEI-Alexa 488-NADH crystals were analyzed by confocal microscopy on a Leica SP8 confocal microscope using the DAPI, Alexa 568, and Alexa 488 channels.

Size distribution of PEI polymers before and after binding to Cry3Aa crystals

To evaluate whether the channels of Cry3Aa crystals affect the size distribution of the PEI molecules that bind within the crystal, dynamic light scattering (DLS) measurements were performed on the PEI polymers before and after incubation with Cry3Aa crystals. For this experiment, 5 mg of dehydrated Cry3Aa crystals were resuspended in 10 mL of PEI solution (50 µg/mL, pH 7.0) and then vortexed to homogenize the solution. Then EDC/NHS cross-linker (1:2 molar ratio) was slowly dropped into the solution while it was gently stirred. After allowing them to react for 5 h, the sample was centrifuged at 15,000 rpm for 10 min to pellet the crystals. The resulting supernatant and the initial PEI solution were then analyzed by DLS.

Retention of NADH on PEI-3A crystals

The capacity of PEI-3A crystals to retain bound NADH was evaluated by incubating NADH-loaded PEI-3A crystals (10 mg PEI-3A, 216 ± 3 nmol NADH) in 2 mL of sodium phosphate buffer (Na-PB) (10 mM, pH 7.0) for 30 min under slow rotation at 10°C. The samples were then centrifuged at 15,000 rpm for 5 min, and the amount of released NADH in the supernatant was measured spectrophotometrically by measuring the absorbance at 340 nm (ε = 6220 M^{-1} cm^{-1}) on a spectrophotometer (Eppendorf BioSpectrometer, Eppendorf AG, Germany). This process was repeated for a total of 20

cycles, after which the samples were resuspended in 1 M Na-PB and vigorously vortexed to release all bound NADH.

Effect of pH on NADH binding to PEI-3A Crystals

To determine the effect of pH on the NADH binding capacity of PEI-3A crystals, the crystals (0.1 mg/mL) were individually incubated in the presence of different concentrations of NADH (0-0.5 mM) in NaPB and ammonia formate buffer (25 mM) at different pH's (6.0-10.0) for 30 min at 10 °C. After centrifuging the samples at 15,000 rpm for 5 min, the maximum NADH binding capacity of PEI-3A crystals was measured spectrophotometrically at 340 nm. NADH solutions at the same pHs were used as controls.

Impact of salt concentration on NADH binding to PEI-3A crystals

PEI-3A-NADH crystals (10 mg, 183 ± 2 nmol NADH) were incubated in 2 mL of Na-PB (25 mM, pH 7.0) with different concentrations of NaCl (0-800 mM), each at 25°C for 30 min. The samples were then centrifuged at 15,000 rpm for 5 min, and the amount of NADH released from the PEI-3A crystals was determined spectrophotometrically by measurement of the A_{340} of the supernatant solution.

NADH release from PEI-3A crystals by mechanical shaking

The potential impact of mechanical agitation on the release rate of NADH molecules from PEI-3A crystals was investigated by resuspending PEI-3A-NADH crystals (10 mg, 2 µmol) in sodium phosphate buffer (Na-PB) (10 mM, pH 7.0) and subjected to incubation at different shaking speeds (0, 400, 800, 1200, 1600, and 2000 rpm) (Eppendorf® ThermoMixer® C, Eppendorf AG, Germany) for 10 min at 25°C. Once the shaking

stopped, the samples were immediately centrifuged at 15,000 rpm for 5 min, and the amount of released NADH was determined spectrophotometrically by measurement of the A_{340}.

Rebinding of NADH molecules into PEI-3A crystals

The rebinding of NADH molecules into PEI-3A crystals was investigated by first shaking the NADH-loaded PEI-3A crystals (10 mg PEI-3A and 2 µmol NADH) in Na-PB buffer (10 mM, pH 7.0) for 10 min at 1000 rpm and 25°C. The crystals were immediately centrifuged at 15,000 rpm for 5 min, and the amount of the released NADH was determined by measuring the A_{340} of the supernatant. The supernatant was returned to the original tube, and the precipitated crystals were resuspended and then incubated for 30 min under gentle rotation at 10 °C. The amount of rebound NADH following this gentle incubation was determined by first centrifuging the sample and then measuring the A_{340} of the supernatant.

NADH binding to PEI-3A crystals as a function of mechanical agitation speed

To determine the K_d and B_{max} constants of NADH binding into PEI-3A crystals at different mechanical shaking conditions, a fixed amount of PEI-3A crystal (0.1 mg) was incubated with different concentrations of NADH (0-1000 µM) at different mechanical shaking conditions (0, 400, 800, 1200, 1600, and 2000 rpm) for 5 min at 25°C. The samples were then subsequently centrifuged at 15000 rpm for 5 min, and the difference in the A_{340} of the supernatant compared to that of the no crystal control was used to determine the amount of NADH bound.

Cry3Aa mediated co-immobilization of GFP and mCherry proteins

The plasmids of pHT315-Cry3Aa-mCherry and pHT315-Cry3Aa-GFP were produced previously[15]. For co-expression of Cry3Aa-mCherry and Cry3Aa-GFP, a pHT315-Cry3Aa-GFP-RBS-Cry3Aa-mCherry plasmid was prepared by first amplifying the *Cry3Aa-mCherry* gene with Kappa HiFi DNA polymerase (Kappa Biosystems) using primers that placed a ribosome binding site (RBS) upstream of the *Cry3Aa-mCherry* gene (**Table S5**). The pHT315-Cry3Aa-GFP plasmid was linearized via digestion with *KpnI*, and then the amplified *rbs-cry3Aa-mCherry* insert was fused downstream of the *Cry3Aa-GFP* gene by Gibson assembly. The plasmid was transferred to the ER2925 strain via heat shock for production of the demethylated plasmid, and then the plasmid was transformed into *Bt* cells via electroporation. The cells were grown in SSM (Schaeffer's sporulation medium) in the presence of erythromycin (50 µg/mL) at 25°C and 220 rpm for 72 h, and then harvested by centrifugation at 8000 rpm. The cells were lyzed via overnight incubation with chicken lysozyme (Sigma) (1 mg/mL) followed by sonication (3 cycles of 2 s on, 2 s off) at 40% power in an ice/water bath. The resulting pellet of Cry3Aa-GFP/Cry3Aa-mCherry particles were subsequently washed with 0.5 M NaCl, 0.25 M NaCl, and ddH_2O and then characterized by fluorescence microscopy (Nikon TE300 microscope). The coefficient values of the Pearson correlation were determined using the software ImageJ.

Construction of Cry3Aa-FDH and Cry3Aa-LDH expression vectors

To construct the expression plasmids of soluble formate dehydrogenase (FDH) and leucine dehydrogenase (LDH) proteins, the *fdh* gene from *Thiobacillus sp. KNK65MA* and

ldh gene from *Bt* were amplified using Kappa HiFi DNA polymerase (Kappa Biosystems) following the standard protocol. Subsequently, the corresponding genes were cloned into a pET28b vector using *XhoI* and *NdeI* restriction sites via the Gibson Assembly Reaction (NEB). This resulted in the incorporation of a C-terminal His-tag for FDH and an N-terminal His-tag for LDH.

To construct the Cry3Aa-FDH and Cry3Aa-LDH expression plasmids, the *fdh* and *ldh* genes were first amplified by PCR reaction and then inserted downstream of the Cry3Aa gene in the pHT315 vector between the *BamHI* and *KpnI* restriction sites using Gibson assembly. For co-expression of Cry3Aa-FDH and Cry3Aa-LDH, a Cry3Aa-FDH/Cry3Aa-LDH co-expression plasmid was produced. Here, the *Cry3Aa-LDH* gene was amplified with primers containing an additional ribosome binding site (RBS), and then the Gibson Assembly Reaction (NEB) was used to insert this gene into a linearized pHT315-Cry3Aa-FDH plasmid at the *KpnI* restriction site. A detailed list of the primers employed for creating the distinct clones can be found in **Table S5.** All DNA modification experiments were conducted using XL10-Gold® ultracompetent cells. Plasmid purification was carried out using the Takara MiniBEST kit 4.0, and the sequence of the plasmids was confirmed by BGI.

Cry3Aa NADH-dependent enzyme fusion crystals

To produce Cry3Aa fusion NADH-dependent enzyme fusion crystals, the pHT315-Cry3Aa-FDH, pHT315-Cry3Aa-LDH, and pHT315-Cry3Aa-FDH/Cry3Aa-LDH plasmids were constructed as described above. Then, they were individually introduced into *Bacillus thuringiensis BT407G* cells via electroporation. The cells were then cultured in

500 mL of SSM and 50 µg/mL of erythromycin at 25°C and 220 rpm for 72 h. Following the cultivation, the cells were collected by centrifugation at 8000 rpm for 8 min using an Avanti J25 Ultracentrifuge (Beckman Coulter). The cell pellets were then resuspended in ddH_2O in the presence of chicken lysozyme (Sigma) (1 mg/mL) for overnight at room temperature, followed by sonication in an ice-water pack for 30 min to achieve complete cell lysis. After that, the particles were purified with 0.5 M NaCl, 0.25 M NaCl, and ddH_2O. The proteins were then characterized using a 10% SDS-PAGE gel. The concertation of the particles was determined using Bradford (Bio-Rad).

Expression and purification of soluble FDH and LDH proteins

To express the FDH and LDH control proteins, pET28b-FDH and pET28b-LDH plasmids were individually transformed into *E. coli* BL21(DE3) cells using heat shock. The grown colonies were then cultured in Lennox Broth (LB, IBI Scientific) in the presence of kanamycin (50 µg/ml) at 37°C. Once the optical density reached 0.6-0.8 at 600 nm (OD600), the protein expression was induced by adding 0.2 mM IPTG, after which the cells were allowed to grow overnight at 25°C. The cells were then harvested by centrifugation at 8,500 rpm for 10 min, and resuspended in a buffer containing 50 mM NaH_2PO_4, 300 mM NaCl (pH 7.0), 1 mM phenylmethylsulfonyl fluoride (PMSF, Cayman Chemicals), and 1 mM benzamidine chloride (TCI Chemicals). The cells were sonicated at 0°C using an ice/water pack for 30 min, followed by centrifugation for 30 min at 8,500 rpm and 4°C. The cell lysis was then filtered and loaded onto nickel resin chromatography (BioRad) for affinity purification. The target proteins were eluted with 250 mM imidazole, and their purities were analyzed using a 12% SDS-PAGE gel.

Identification of L-*tert*-Leu by HPLC

Derivatization of L-*tert*-Leu with o-phthalaldehyde (OPA) enables its quantitation by fluorescence. Thus, after combining the reaction supernatants with carbonate buffer (500 mM, pH 10.5, 10% v/v), the solutions were incubated with OPA (2 mg/mL) for 1 minute before being injected into an HPLC for analysis. Reverse-phase HPLC was conducted using a C18 column (10 μm, 4.6 × 250 mm, Agilent) at 25°C, with a flow rate of 1 mL/min and the excitation and emission wavelengths of the fluorescent detector set to 340 nm and 455 nm, respectively. The elution was performed using a methanol gradient (0-100%, 5% increase per min).

Determining the chirality of the *tert*-leucine product

To determine the chirality of the *tert*-Leu products produced by Cry3Aa-FDH/Cry3Aa-LDH particles, the reaction products were mixed with a carbonate buffer (500 mM, pH 10.5) at a concentration of 10% v/v, and subsequently mixed with Marfey's reagent, 1-fluoro-2,4-dinitrophenyl-5-L-alanine amide, and incubated for 90 min at 40°C. Before analysis, the mixture was diluted with methanol. The analysis was performed using gradient elution in a reverse-phase HPLC system equipped with a C18 column (10 μm, 4.6 × 250 mm, Agilent) for the chiral detection of L- and D-*tert*-Leu derivatives. The elution was performed using a methanol gradient (0-100%, 5% increase per min) over a span of 20 min with the modified L- and D-*tert*-leu derivatives produced, monitored by their absorbance at 340 nm.

Determination of enzyme kinetic parameters

For the determination of kinetic parameters for L-*tert*-Leu production, the activities of LDH (3 µg/mL) and Cry3Aa-FDH/Cry3Aa-LDH particles (100 µg/mL) were assessed at various concentrations of TMP (0-30 mM) but a fixed concentration of NADH (0.3 mM). Lineweaver-Burk plots were drawn and analyzed to calculate K_m and V_m values for TMP.

Similarly, the kinetic parameter for formic acid oxidation was studied for FDH (3 µg/mL) and Cry3Aa-FDH/Cry3Aa-LDH particles (100 µg/mL) at different formic acid concentrations ranging from 0 to 50 mM while maintaining a fixed concentration of 0.3 mM NAD^+. All the reactions were initially stopped with 70% methanol, and then assayed by determining the amount of NADH produced based on the A_{340}. One unit of oxidation activity was defined as the amount of enzyme required to produce 1 µmol of NADH per minute at 25°C. Similarly, one unit of reduction activity was defined as the amount of enzyme needed to produce 1 µmol of NAD^+ per minute.

Effect of mechanical shaking on L-*tert*-Leu production efficiency

To understand the potential role of mechanical agitation on L-*tert*-Leu production, 106 mM of Cry3Aa-FDH/Cry3Aa-LDH particles were prepared in reaction buffer containing, ammonia formate (30 mM, pH 8.0), TMP (25 mM), and 0.5 mM NADH, and then incubated at 25°C in a thermomixer with (1000 RPM) and without (0 RPM) mechanical agitation at 25 °C for 2 h. The samples were then centrifuged and analyzed using HPLC.

Stability of Cry3Aa-FDH /Cry3Aa-LDH particles under mechanical agitation

To explore the stability of the Cry3Aa-FDH/Cry3Aa-LDH particles under mechanical shaking, we incubated Cry3Aa-FDH and Cry3Aa-LDH particles (12 mg/mL) with and

without mechanical shaking (0 and 1000 rpm) in ammonia formate buffer (30 mM and pH:8.0) at 25 °C for 21 days, and then the activity of both FDH and LDH components was separately measured every week. To measure the activity of the FDH component, the particles (0.1 mg/mL) were incubated in the presence of reaction buffer containing 25 mM sodium formate and 0.5 mM NAD^+ in NaPB (30 mM, pH 8.0) for 30 min at 25°C and 1000 rpm, and the absorption of NADH was monitored spectroscopically at 340 nm. Similarly, the activity of the LDH component was measured at 340 nm by incubation of the particles (0.1 mg/mL) in reaction buffer containing 25 mM TMP and 0.5 mM NADH in ammonia formate (30 mM, pH 8.0) for 30 min at 25 °C and 1000 rpm. The activity of FDH and LDH components was considered to be 100% for the first day. Finally, a SEM study was conducted to examine the morphology of Cry3Aa-FDH and Cry3Aa-LDH particles with and without shaking after three weeks. 5 µL of the particles in ddH_2O were dropped onto a carbon glass and kept at room temperature for overnight drying. The samples were then coated with an 80/20 mixture of Pd and Pt using a Q150T ES turbomolecular pump coater before imaging.

Combining PEI-3A-NADH and Cry3Aa-FDH/Cry3Aa-LDH particles for recyclable L-*tert*-leu biosynthesis

To explore the recyclability and retention of NADH in a coupled system with NADH-loaded PEI-3A and Cry3Aa-FDH/Cry3Aa-LDH crystals for the biosynthesis of L-*tert*-leucine (L-*tert*-Leu). 12 mg/mL of PEI-3A crystals were initially incubated in the presence of NADH (0.5 mM) in ddH_2O under slow rotation at 10°C for 10 min. Then, the amount of bound NADH was quantified based on the A_{340}. After several washings of the crystals

with ddH_2O, PEI-3A crystals loaded with 0.5 mM NADH were mixed with Cry3Aa-FDH/Cry3Aa-LDH particles (12 mg/mL) in a reaction buffer containing 30 mM ammonia formate (pH 8.0) and TMP (25 mM). Then, the samples were subjected to mechanical shaking (1000 rpm) for 23.5 h at 25 °C. After the reaction was complete, the samples were incubated at 10°C for 30 min to complete the NADH rebinding step, and then centrifuged at 7,000 rpm for 7 min to pellet the particles and crystals before collecting the supernatants for HPLC analysis. Then, fresh reaction buffer was added to the samples to resuspend the precipitated crystals and particles and start the next reaction cycle. The Cry3Aa-FDH/Cry3Aa-LDH particles only (i.e. no PEI-3A) were treated similarly, except that 0.5 mM NADH was added at the start of each cycle. This process was repeated for 21 cycles.

Determination of the L-*tert*-Leu produced by different LDH and FDH constructs

The synthesis of L-*tert*-Leu was investigated using a soluble mixture of FDH+LDH proteins, and their corresponding Cry3Aa fusion particles. In this experiment, 50 mM of TMP and 0.5 mM of NADH were dissolved in 100 mM of ammonia formate buffer at a pH of 8.0. Then, 30 µmole/mL of FDH+LDH (1:1), Cry3Aa-FDH+Cry3Aa-LDH (1:1), and Cry3Aa-FDH/Cry3Aa-LDH particles were added. The mixtures were maintained for 24 h under shaking conditions at 1000 rpm and 25°C. Following centrifugation, the supernatants were collected for subsequent analysis using HPLC.

Scanning electron microscopy (SEM) of Cry3Aa-FDH/Cry3Aa-LDH particles

The size, structure, and morphology of Cry3Aa-FDH/Cry3Aa-LDH particles were examined using scanning electron microscopy (SEM). For the analysis, 5 µL of the

particles in ddH_2O were deposited onto a carbon glass substrate and allowed to dry slowly overnight. The dried samples were then coated with an 80/20 mixture of Pd/Pt using a Q150T ES turbomolecular pump coater.

Production of PEI-modified mesoporous silica beads

PEI-modified silica beads with different bead and pore sizes, including PEI-SB1 (1 µm beads with 4 nm pores), PEI-SB2 (1 µm beads with 10 nm pores), and PEI-SB3 (150 nm size with 4 nm pores), were modified with (3-Aminopropyl)trimethoxysilane (APTMS) to place amino groups on the bead surface and channels[16, 17]. This step was achieved by immersing the beads in a 0.2% APTMS solution in anhydrous toluene under a vacuum for 15 h, washing with a mixture of toluene and methanol (50% v/v) to remove residual methanol, and then drying at 80°C for 4 h. The amino-functionalized silica beads were subjected to gentle rotation and immersed in a solution of glutaraldehyde (0.5% wt in PBS, 100 mM, pH 7.0) for 30 minutes at 25°C, and then a PEI solution (0.6 mg/mL) was added to the functionalized beads in a PBS buffer (100 mM, pH 7.0) and allowed to rotate gradually for 2 h at 25°C. To block any remaining reactive glutaraldehyde groups, the beads were flushed with ethanolamine-HCl (0.5 M, pH 8.5). To determine the amount of PEI bound to silica beads, a standard curve was initially generated by measuring the A_{285} for a series of PEI solutions (0-50 µg/ml) with a fixed amount of 130 mM of Cu(II) sulfate at pH 7.0. The supernatant obtained following the coupling reaction was treated with 130 mM of copper (II) sulfate, and then the absorbance at 285 nm was measured. The amount of PEI remaining was then quantified based on A_{285} and the standard curve. The amount of PEI anchored to the silica beads was determined based on the difference between the

initial amount of PEI added and the amount of PEI remaining in the solution following incubation with the silica beads.

To validate the binding of PEI to the silica beads, Alexa Fluor™ 488-labelled PEI (PEI-Alexa 488) was used in lieu of PEI for binding to the amino-functionalized micrometer-sized silica beads (i.e. PEI-SB1 and PEI-SB2) described above. The resulting PEI-Alexa 488-modified silica beads were washed multiple times with NaCl (200 mM) and then with ddH_2O. Subsequently, they were incubated in the presence of 2.0 mM NADH in ddH_2O for 30 minutes at 10°C with gentle rotation, followed by centrifugation at 8000 rpm for 10 min. The residual NADH in the solution was quantified by measuring the absorbance at 340 nm, followed by the collection of beads for confocal microscopy analysis after serval washing with ddH_2O.

To measure and compare their NADH binding capacities, PEI-modified silica beads (PEI-SB1, PEI-SB2, and PEI-SB3) with the same amount of bound PEI (~ 25 mg/g of SB) were prepared as described above. The beads (2 mg) were then separately incubated in the presence of NADH (2 mM) in ddH_2O for 30 min at 10 °C under slow rotation. The samples were then centrifuged at 8000 rpm for 10 min, and then A_{340} was measured to determine the NADH concentration. The amount of NADH bound to the beads was determined by the difference between NADH initially in solution and after incubation with the beads. These data were then used to determine the maximum NADH binding capacity of PEI-modified silica beads based on their dried weights, which are listed as µmol of NADH / mg of the support.

References

(1) Semenova, A.; Giles, L. W.; Vidallon, M. L. P.; Follink, B.; Brown, P. L.; Tabor, R. F. Copper-Binding Properties of Polyethylenimine-Silica Nanocomposite Particles. *Langmuir.* **2022,** *38* (34), 10585-10600.

(2) Heater, B. S.; Yang, Z.; Lee, M. M.; Chan, M. K., *In Vivo* Enzyme Entrapment in a Protein Crystal. *J. Am. Chem. Soc.* **2020,** *142* (22), 9879-9883.

(3)Yang, Z.; Zheng, J.; Chan, C.-F.; Wong, I. L. K.; Heater, B. S.; Chow, L. M. C.; Lee, M. M. M.; Chan, M. K. Targeted Delivery of Antimicrobial Peptide by Cry Protein Crystal to Treat Intramacrophage Infection. *Biomater.* **2019,** *217*, 119286.

(4) Mounsef, J. R.; Salameh, D.; Awad, M.; Chamy, L.; Brandam, C.; Lteif, R. A Simple Method for the Separation of *Bacillus thuringiensis* Spores and Crystals. *J. Microbiol. Methods.* **2014,** *107*, 147-9.

(5)Ungaro, F.; De Rosa, G.; Miro, A.; Quaglia, F. Spectrophotometric Determination of Polyethylenimine in the Presence of an Oligonucleotide for the Characterization of Controlled Release Formulations. *J.Pharm.Biomed. Anal.* **2003,** *31* (1), 143-149.

(6) Wen, T.; Qu, F.; Li, N. B.; Luo, H. Q., A Facile, Sensitive, and Rapid Spectrophotometric Method for Copper(II) Ion Detection in Aqueous Media using Polyethyleneimine. *Arab. J. Chem.* **2017,** *10*, S1680-S1685.

(7) Velasco-Lozano, S.; Benítez-Mateos, A. I.; López-Gallego, F. Co-immobilized Phosphorylated Cofactors and Enzymes as Self-Sufficient Heterogeneous Biocatalysts for Chemical Processes. *Angew. Chem., Int. Ed. Engl.* **2017,** *56* (3), 771-775.

(8) Yang, Z.; Lee, M. M. M.; Chan, M. K. Efficient Intracellular Delivery of p53 Protein by Engineered Protein Crystals Restores Tumor Suppressing Function *In Vivo*. *Biomater.* **2021,** *271*, 120759.

(9) Otwinowski, Z.; Minor, W. Processing of X-ray Diffraction Data Collected in Oscillation Mode. *Methods Enzymol.* **1997,** *276*, 307-26.

(10) McCoy, A. J.; Grosse-Kunstleve, R. W.; Adams, P. D.; Winn, M. D.; Storoni, L. C.; Read, R. J. Phaser Crystallographic Software. *J. Appl. Crystallogr.* **2007,** *40* (4), 658-674.

(11) Winn, M. D. An Overview of the CCP4 Project in Protein Crystallography: An Example of a Collaborative Project. *J. Synchrotron Radiat.* **2003,** *10* (1), 23-5.

(12) Emsley, P.; Cowtan, K. Coot: Model-building Tools for Molecular Graphics. *Acta Crystallogr. Sect D: Biol. Crystallogr.* **2004,** *60* (12), 2126-32.

(13) Murshudov, G. N.; Skubák, P.; Lebedev, A. A.; Pannu, N. S.; Steiner, R. A.; Nicholls, R. A.; Winn, M. D.; Long, F.; Vagin, A. A. REFMAC5 for the Refinement of Macromolecular Crystal Structures. *Acta Crystallogr. Sect D: Biol. Crystallogr.* **2011,** *67* (4), 355-67.

(14) Liebschner, D.; Afonine, P. V.; Baker, M. L.; Bunkóczi, G.; Chen, V. B.; Croll, T. I.; Hintze, B.; Hung, L. W.; Jain, S.; McCoy, A. J.; *et al.* Macromolecular Structure Determination using X-rays, Neutrons and Electrons: Recent Developments in Phenix. *Acta Crystallogr. Sect D: Biol. Crystallogr.* **2019,** *75* (10), 861-877.

(15) Nair, M. S.; Lee, M. M.; Bonnegarde-Bernard, A.; Wallace, J. A.; Dean, D. H.; Ostrowski, M. C.; Burry, R. W.; Boyaka, P. N.; Chan, M. K. Cry Protein Crystals: A Novel Platform for Protein Delivery. *PLOS One.* **2015,** *10* (6), e0127669.

(16)Goscianska, J.; Olejnik, A.; Nowak, I. APTES-Functionalized Mesoporous Silica as a Vehicle for Antipyrine – Adsorption and Release Studies. *Colloids Surf. A: Physicochem. Eng. Asp.* **2017,** *533*, 187-196.

(17) Bourkaib, M. C.; Gaudin, P.; Vibert, F.; Guiavarc'h, Y.; Delaunay, S.; Framboisier, X.; Humeau, C.; Chevalot, I.; Blin, J.-L. APTES Modified SBA15 and Meso-Macro Silica Materials for the Immobilization of Aminoacylases from *Streptomyces ambofaciens*. *Microporous Mesoporous Mater.* **2021,** *323*, 111226.
